# Unraveling surface sensitivity for generating metastable active sites in molybdenum-based catalysts for CO_2_ hydrogenation

**DOI:** 10.1038/s41467-025-66430-3

**Published:** 2025-11-24

**Authors:** Yifan Feng, Zhenyu Xing, Daoping Ye, Jin Niu, Yu Tian, Tian Ma, Chong Cheng, Bo Yin, Arne Thomas, Shuang Li

**Affiliations:** 1 College of Polymer Science and Engineering, State Key Laboratory of Advanced Polymer Materials, Sichuan University, Chengdu, China; 2 College of Chemical Engineering, Sichuan University, Chengdu, China; 3 Functional Materials, Department of Chemistry, Technische Universität Berlin, Berlin, Germany

**Keywords:** Heterogeneous catalysis, Catalyst synthesis, Porous materials

## Abstract

The reverse water-gas shift (RWGS) reaction is crucial for sustainable CO_2_ conversion, yet catalyst surface remodeling at high temperatures remains a complex and pivotal phenomenon. This study investigates the complex relationship between surface reconstruction and catalytic performance using a series of molybdenum-based catalysts, which can generate different catalytic MoO_3_ surface layers under RWGS conditions. In-situ characterization techniques and theoretical analyses reveal that the MoO_3_ layer on MoO_3_/MoO_2_-C and MoO_3_/Mo_2_N-C is in-situ reduced to MoO_2_ and metastable MoO_x_ (2 <x < 3), respectively, while it is not reduced on MoO_3_/Mo_2_C-C during the catalysis process. The metastable MoO_x_ species on MoO_3_/Mo_2_N-C shows an unprecedented CO yield (up to 48.3 %) nearing the equilibrium conversion limit, a CO formation rate of 8.26 × 10^−5^ mol_CO_ g_cat_^‒1^ s^‒1^, and 99 % CO selectivity under 500 °C. The function and formation conditions of metastable MoO_x_ sites are comprehensively investigated in this work.

## Abstract (web summary)

Reverse water-gas shift is key for CO₂ use, but high-temperature surface reconstruction obscures structure-performance links. Here, metastable MoO_x_ on Mo_2_N gives equilibrium-level CO_2_ conversion and 99% CO selectivity at 500 °C.

## Introduction

Thermal catalytic reduction of CO_2_ into value-added products has been considered one of the most promising strategies for achieving carbon-neutralization goals^1–3^. Worldwide initiatives are aimed at establishing a sustainable hydrogen infrastructure^4–6^, which has positioned the hydrogenation of carbon dioxide as a viable route for CO_2_ utilization^7–9^. However, due to the stability of CO_2_ molecules, the transformation of CO_2_ usually relies on highly active heterogeneous catalysts^10^. The production of carbon monoxide (CO) via the reverse water gas shift reaction (RWGS) is a reaction of immense economic and ecological importance. This is because the resulting CO or synthesis gas (a mixture of CO and H_2_) can serve as fundamental components for synthesizing a wide array of chemicals within sequential catalytic processes^11^. Economic analyses suggest that pathways that use CO_2_ to generate CO could offer competitive advantages over other methods, especially in scenarios where renewable energy sources are abundant^12,13^. This underscores the strategic importance of developing and optimizing RWGS reactions and their integration into the broader chemical production industry^14–17^.

To fully harness the potential of the RWGS reaction on a global scale, the catalyst development needs to meet important criteria^18–20^. For instance, high CO selectivity is required to prevent hydrogen from being lost to methane or other side-products, which simplifies the downstream separation process^21–23^. However, due to the endothermic nature of the reaction, relatively high operating temperatures, of up to 800 °C, and ideally high H_2_/CO_2_ ratios are required to achieve a satisfactory conversion^10^. Such reaction conditions often lead to sintering and agglomeration of the catalysts, particularly with commonly used high-content Cu- or noble metal-based catalysts, ultimately resulting in poor stability during long-term practical use^24–27^. Recently, numerous efforts have been devoted to developing new catalyst systems for RWGS reactions with high activity and stability. In this regard, some progress has also been achieved for Mo-based catalysts. For example, MoP supported on CNTs has been reported for RWGS. While this catalyst has a low CO_2_ conversion rate of only 16% it showed 100% CO selectivity at 500 °C under the weight hourly space velocity (WHSV) of 36,000 mL g_cat_^‒1^ h^‒1^ ^28^. Single-atom molybdenum supported on nitrogen-doped carbon (Mo/NC) has also been investigated as RWGS catalyst, achieving a CO_2_ conversion rate of 30.4% with nearly 100% CO selectivity at 500 °C, even under extremely low H_2_ partial pressure^29^. Recently, nanocrystalline cubic molybdenum carbide (α-Mo_2_C) has demonstrated remarkable performance, achieving approximately 60% CO_2_ conversion and 100% CO selectivity at high space velocity, even after over 500 h of exposure to harsh reaction conditions at 600 °C^30^. These studies indicate that Mo-based materials are very promising catalysts for RWGS in practical applications. However, the performance of different Mo components differs a lot when their supports, structures, synthesis conditions, and reaction conditions change^2,31–33^. This poses a great challenge to the reasonable design of high-performance Mo-based catalysts and a precise understanding of its mechanism, especially under high temperatures, thereby hindering the further development of Mo-based RWGS catalysts. We have recently reported that organic-polyoxometallate precursors form molybdenum nitrides or carbides, depending on the carbonization temperature^32^. This precursor offers the possibility of investigating and solving the challenges mentioned above. Herein, we designed a series of catalytic MoO_3_ surface layers on different molybdenum catalysts from an organic-polyoxometalate crystal precursor, prepared via condensation of ammonium molybdate and p-phenylenediamine, as a system for a comprehensive investigation of the catalytic mechanisms and the reconstruction behavior of metastable active sites under RWGS conditions. By adjusting the carbonization temperatures, the organic-polyoxometalate precursor transformed into MoO_2_, Mo_2_N, and Mo_2_C nanocrystals on a carbon matrix, named MoO_2_-C, Mo_2_N-C, and Mo_2_C-C. When exposed to air during the synthesis process, a uniform surface oxidization layer of MoO_3_ is formed on these catalysts (i.e., MoO_3_/MoO_2_-C, MoO_3_/Mo_2_N-C, and MoO_3_/Mo_2_C-C), therefore providing materials which allow the systematic investigation of the surface and bulk catalytic properties of different Mo compounds. Comprehensive in-situ characterization techniques and theoretical analyses revealed that the MoO_3_ layer on MoO_3_/MoO_2_-C and MoO_3_/Mo_2_N-C will be in-situ reduced to MoO_2_ and metastable MoO_x_ (2 < x < 3), respectively, while it is not reduced on MoO_3_/Mo_2_C-C during the catalysis process. The activity of these catalysts for the RWGS reaction has been investigated, which indicates that the metastable MoO_x_ (2 < x < 3) species on MoO_3_/Mo_2_N-C shows an unprecedented CO_2_ conversion (up to 48.3 %) close to the equilibrium conversion limit, a CO formation rate of 8.26 × 10^−5^ mol_CO_ g_cat_^‒1^ s^‒1^, and up to 99% CO selectivity at 500 °C. In this work, the formation conditions of metastable MoO_x_ active sites and their function for catalysis in RWGS have been comprehensively investigated. The strategy of constructing highly active metal oxide surfaces by adjusting the reducibility of the substrate can provide new insights into the design of high-performance heterogeneous catalysts for various reactions, such as the RWGS and Fischer–Tropsch processes.

## Results

### Structure characterization of molybdenum-based catalysts

**Fig. 1:**
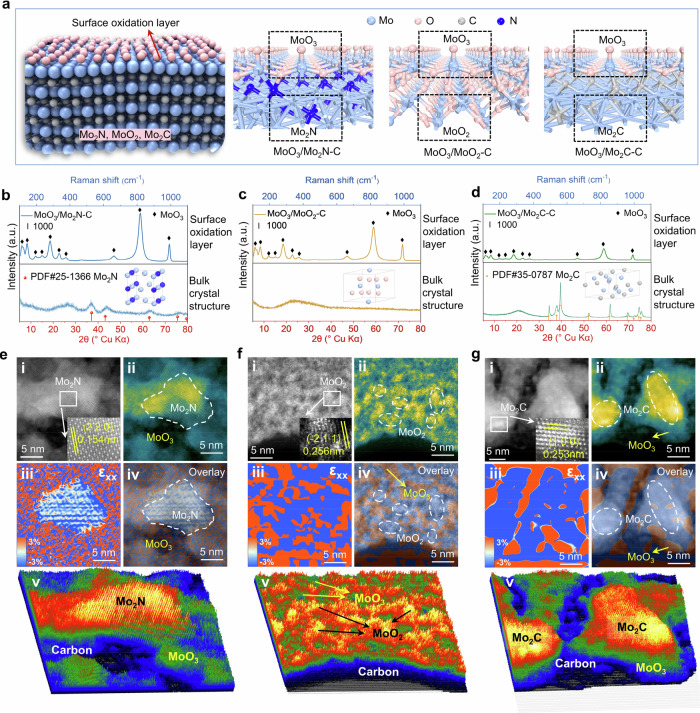
Characterization of the heterostructured molybdenum-based catalysts. **a** Schematic illustration of the catalyst’s surface structure. Light blue: molybdenum atoms; dark blue: nitrogen atoms; pink: oxygen atoms; gray: carbon atoms. **b**–**d** XRD patterns and Raman spectra. The inset shows the model schematic diagram of Mo_2_N, MoO_2_, and Mo_2_C; **e**–**g** HAADF-STEM images and elucidation of the heterojunctions in MoO_3_/Mo_2_N-C, MoO_3_/MoO_2_-C, and MoO_3_/Mo_2_C-C. i) HAADF-STEM images; ii) false color image of the HAADF-STEM image from i); iii, iv) lattice strain map of MoO_3_/Mo_2_N-C, MoO_3_/MoO_2_-C, and MoO_3_/Mo_2_C-C from i); v) 3D surface plot of MoO_3_/Mo_2_N-C, MoO_3_/MoO_2_-C, and MoO_3_/Mo_2_C-C from i).

To verify that the as-prepared catalysts have the expected structure as shown in Fig. 1a, comprehensive characterizations were carried out. Scanning electron microscopy (SEM) measurements were first conducted to reveal the microscale morphologies of the as-synthesized catalysts. As shown in Supplementary Fig. 1, the uniform nanosheet structures from the metal-organic precursors have been maintained in the pyrolyzed samples. The surface oxidation structure was probed using Raman spectroscopy, showing characteristic MoO_3_ peaks present across all three catalysts. Notably, MoO_3_/Mo_2_N-C exhibits the most intense peaks, indicative of a higher MoO_3_ content on the Mo_2_N surface (Fig. 1b–d). Powder X-ray diffraction (XRD) was used to describe the crystallographic characteristics of the catalysts. The MoO_3_/Mo_2_N-C and MoO_3_/Mo_2_C-C exhibit distinct diffraction peaks aligning with the known patterns of Mo_2_N (PDF no. 25-1366) and Mo_2_C (PDF no. 44-1159) (Fig. 1b–d). Conversely, the MoO_3_/MoO_2_-C displays just a broad peak around 25°, suggesting the mainly amorphous nature of the MoO_2_ component with only small crystalline areas (vide infra).

Further investigation using high-angle annular dark-field scanning transmission electron microscopy (HAADF-STEM) illuminates the consistent nano-sized crystal arrangement interconnected by the carbon substrate, forming a coherent 2D nanosheet structure. Crystalline structures are discerned with clear lattice fringes corresponding to the planes of Mo_2_N (220), MoO_2_ (−211), and Mo_2_C (100), with respective spacings of 0.154, 0.256, and 0.253 nm, which confirms the precise control over the bulk phase of the catalysts (Fig. 1e–g_i_, Supplementary Figs. 2–4). Based on the corresponding HAADF images with false color and the surface plot (Fig. 1e–gii, v), the thickness distribution on the surfaces of the three catalysts can be obtained, which allows for a preliminary assessment of the distribution of Mo_2_N, MoO_2_, Mo_2_C, and MoO_3_ on the three catalysts. To obtain more accurate distribution information, geometric phase analysis (GPA) was further conducted on the MoO_3_/Mo_2_N-C, MoO_3_/MoO_2_-C, and MoO_3_/Mo_2_C-C catalysts (Fig. 1e–giii). The strain tensor component ε_xx_ reveals that lattice strain exists in all three catalysts, which is a result of the interactions between Mo_2_N, MoO_2_, Mo_2_C, and MoO_3_ (Red represents tensile stress, while blue indicates compressive stress). Due to these interactions, the intrinsic stress of Mo_2_N, MoO_2_, and Mo_2_C differs from that of MoO_3_. The distribution of molybdenum species can be more accurately determined by combining the results from Fig. 1e–gi-iii as shown in the overlay in Fig. 1e–giv and the 3D surface plots shown in Fig. 1e–gv. Elemental mapping via energy-dispersive X-ray spectroscopy (EDS) from HAADF-STEM provide evidence of the uniform distribution of Mo, N, O, and C across the materials, as visualized in Supplementary Figs. 5–7.

**Fig. 2:**
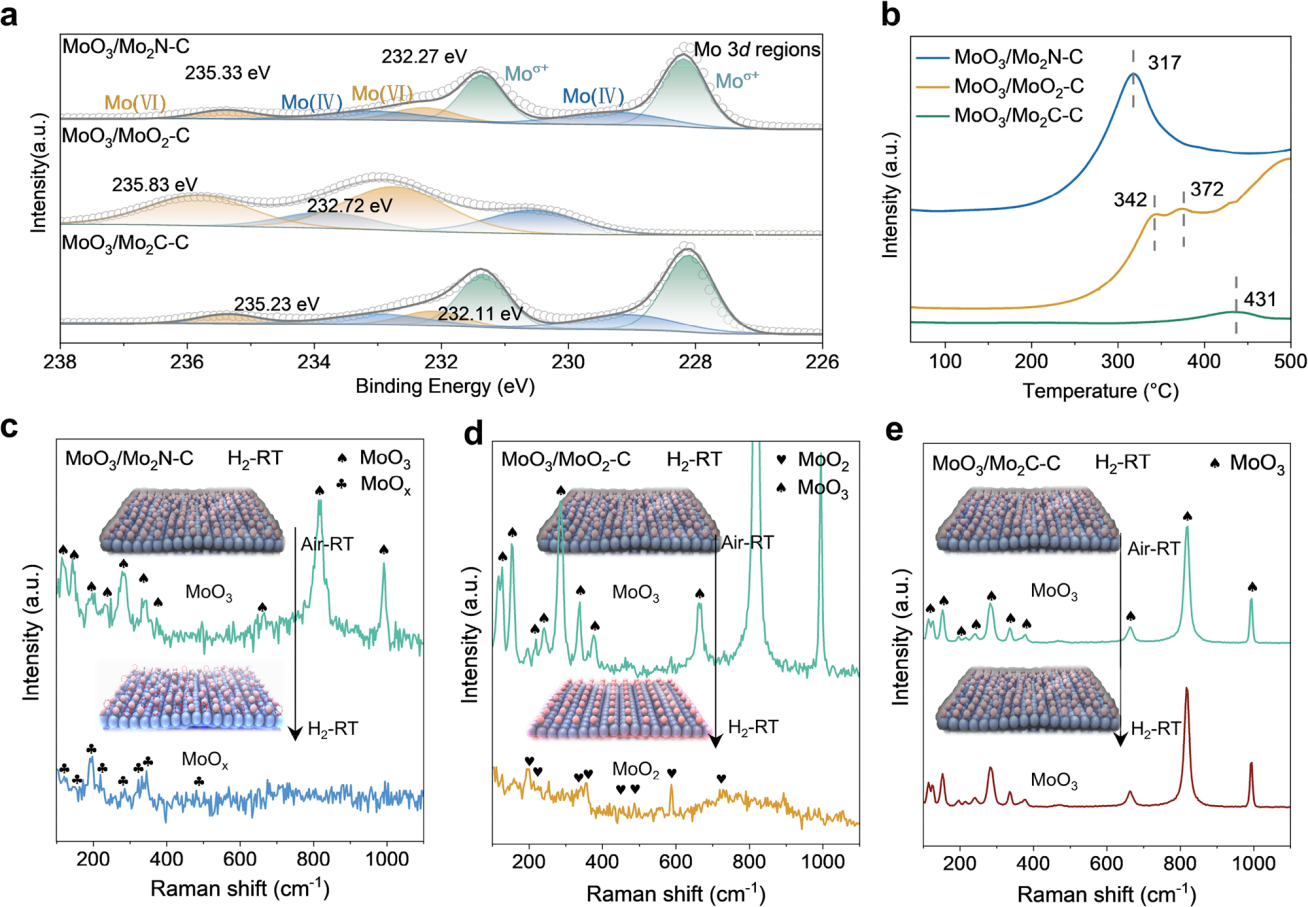
Electronic structure analysis and surface reconstruction mechanism investigation of the MoO_3_/Mo_2_N-C, MoO_3_/MoO_2_-C, and MoO_3_/Mo_2_C-C catalysts. **a** Curve-fitted high-resolution XPS Mo 3*d* spectra of the different materials. **b** H_2_-TPR of MoO_3_/Mo_2_N-C, MoO_3_/MoO_2_-C, and MoO_3_/Mo_2_C-C catalysts. **c**–**e** In-situ Raman results of the materials under H_2_ pretreatment. The inset shows schemes of the surface structure of three catalysts in different atmospheres.

X-ray photoelectron spectroscopy (XPS) was conducted to probe the electronic configuration and oxidation states of the MoO_3_/Mo_2_N-C, MoO_3_/MoO_2_-C, and MoO_3_/Mo_2_C-C catalysts (Supplementary Figs. 8, 9). Figure 2a presents the high-resolution Mo 3*d* spectra of these catalysts, where MoO_3_/Mo_2_N-C and MoO_3_/Mo_2_C-C show apparent peaks of Mo^δ+^ at 228.8 and 231.9 eV, indicating the existence of Mo_2_N and Mo_2_C as main components. In contrast, in MoO_3_/MoO_2_-C, only the peaks for Mo^4+^ (MoO_2_, 229.4 and 233.2 eV) and Mo^6+^ (MoO_3_, 232.7 and 235.8 eV) can be observed, indicating that the stoichiometric Mo oxides are the main component for MoO_3_/MoO_2_-C. All three catalysts exhibit distinct Mo^6+^ peaks, confirming the presence of surface MoO_3_ on each material. This result aligns consistently with the Raman characterization data.

The reducibility of the MoO_3_ surface layer on the different substrates has been assessed by temperature-programmed reduction in H_2_ (H_2_-TPR). A pronounced H_2_ uptake peak for MoO_3_/Mo_2_N-C catalyst at 317 °C is noticeable, significantly lower than the peaks at 342 °C for MoO_3_/MoO_2_-C and 431 °C for MoO_3_/Mo_2_C-C, as illustrated in Fig. 2b. The more intense and lower-temperature reduction peak for MoO_3_/Mo_2_N-C implies easier reducibility of surface MoO_3_, conducive to form an active surface layer. Additionally, a secondary peak at 372 °C for MoO_3_/MoO_2_-C suggests a deeper reduction step. This may involve the reduction of MoO_3_ on the MoO_3_/MoO_2_-C surface to MoO_x_ under an H_2_ atmosphere, followed by its subsequent reduction to MoO_2_^34^.

We furthermore systematically examined the surface structural evolution under reductive conditions through in situ Raman spectroscopy. As shown in Fig. 2c, after the H_2_ treatment of the MoO_3_/Mo_2_N-C catalyst at room temperature, the surface MoO_3_ is converted to MoO_x_. It can be assumed that under an H_2_ atmosphere, some lattice oxygen is released from the MoO_3_ on the surface of MoO_3_/Mo_2_N-C, forming MoO_x_. Due to the interactions between MoO_3_ and Mo_2_N, MoO_3_ can be reduced by H_2_, but not sufficient to be fully reduced to MoO_2_, resulting in a phase where 2 < x < 3^35^. Changing the atmosphere again to air at room temperature lead to the return of the surface MoO_x_ phase back to MoO_3_, highlighting the pronounced sensitivity of the surface MoO_3_ on the Mo_2_N substrate to ambient O_2_ or reductive H_2_ conditions, as corroborated by Supplementary Fig. 10, showing the Raman spectra when the atmosphere is changed from H_2_ to air. Conversely, on the MoO_3_/MoO_2_-C catalysts, in-situ H_2_ treatment at room temperature exclusively yields MoO_2_ peaks, signifying that the metastable MoO_x_ species could not be stabilized, and the MoO_3_ is further reduced to MoO_2_ on the MoO_2_ substrate, as depicted in Fig. 2d. Note that in comparison to MoO_3_/Mo_2_N-C, only for MoO_3_/Mo_2_C-C the characteristic peaks of MoO_3_ can be observed in the in situ Raman spectra(Fig. 2e).

**Fig. 3:**
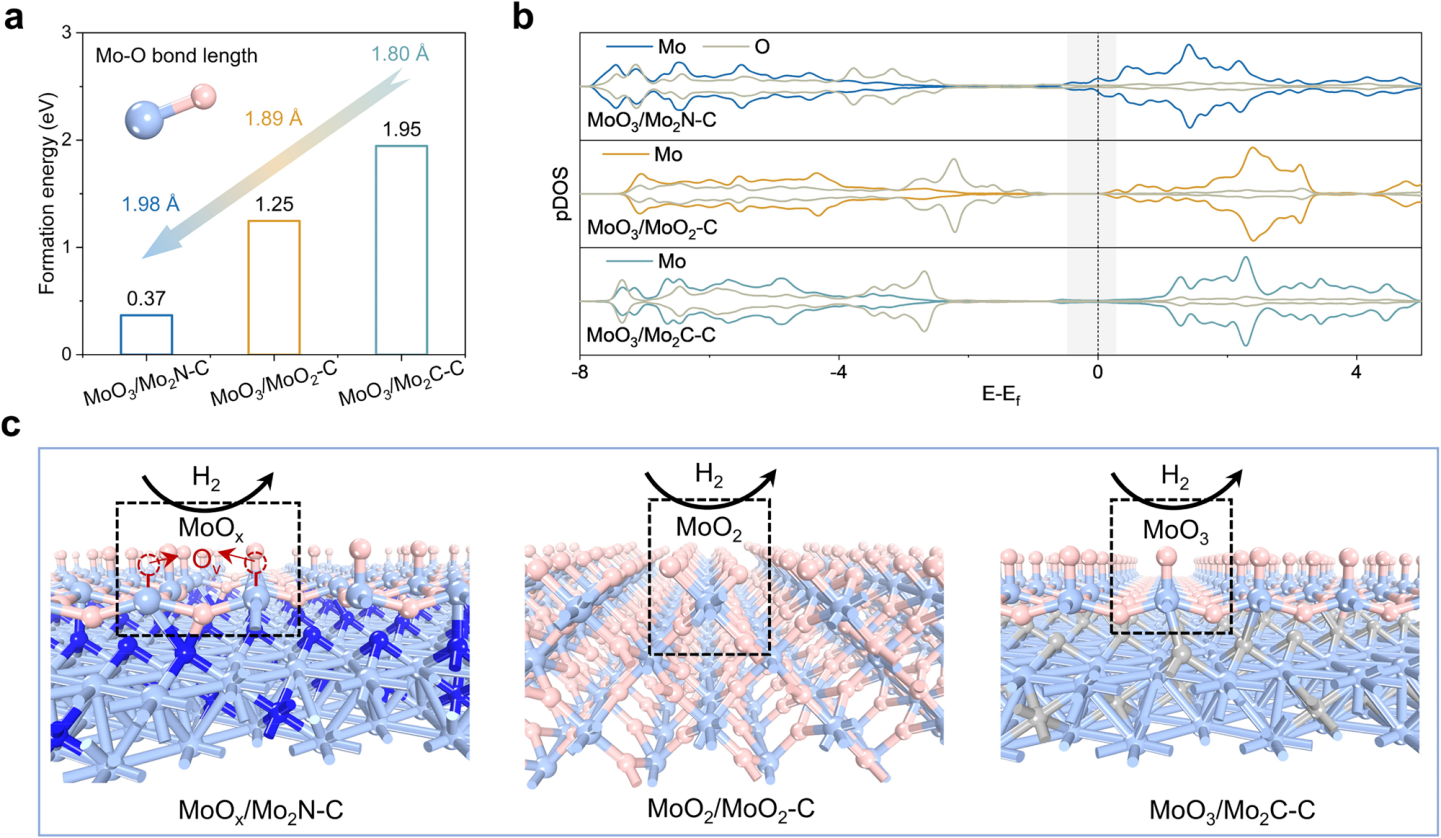
DFT calculations reveal the intrinsic mechanism of oxygen vacancy formation. **a** Calculated formation energy of oxygen vacancy and Mo-O (MoO_3_) bond length of different materials. **b** PDOS analysis of the Mo-O (MoO_3_) bond for different materials. **c** The structural schematic diagrams after surface reconstruction under flowing H_2_.

Furthermore, density functional theory (DFT) calculations were performed to gain deeper insights into the underlying mechanisms of the surface reconstruction phenomenon observed in the in-situ experiments. As shown in Supplementary Fig. 11, the obvious orbital overlap in the partial density of states (pDOS) plots reveals a strong interaction between the substrate (MoO_2_, Mo_2_N, Mo_2_C) and surface oxide layer (MoO_3_) in all cases. The calculated formation energy of an oxygen vacancy in Fig. 3a indicates that MoO_3_/Mo_2_N-C own a much lower energy barrier (0.37 eV) to lose oxygen from the oxide layer when compared with MoO_3_/MoO_2_-C (1.25 eV) and MoO_3_/Mo_2_C-C (1.95 eV), which is consistent with the sequence observed in the H_2_-TPR results, indicating that Mo₂N more readily forms oxygen vacancies in supported MoO_3_. When the substrate (Mo_2_N, MoO_2_, Mo_2_C) comes into contact with the surface layer (MoO_3_), different electronic interactions arise, thereby influencing the properties of the surface layer. The calculation results show that in MoO_3_/Mo_2_N-C, the Mo-O bonds in the surface MoO_3_ layer are significantly elongated with a value of 1.98 Å resulting from the strong interaction between Mo_2_N and MoO_3_, weakening the strength of the Mo-O bonds; while MoO_3_/MoO_2_-C and MoO_3_/Mo_2_C-C exhibit a relatively shorter Mo-O bond length of 1.89 Å and 1.80 Å, respectively (insets in Fig. 3a). Moreover, the pDOS plots in Fig. 3b indicate that the MoO_3_ layer in MoO_3_/Mo_2_N-C exhibits a relatively prominent electronic state near the Fermi level, which facilitates electron transfer during the reaction and promotes the formation of oxygen vacancies, as further supported by the oxygen vacancy formation energy calculations shown in Fig. 3a. However, in MoO_3_/MoO_2_-C and MoO_3_/Mo_2_C-C, the strong Mo–O bonds and limited surface electron transfer, reflected by their larger band gaps, make the formation of MoO_x_ species more difficult. Surface reconstruction under flowing hydrogen conditions is shown in Fig. 3c.

### Reactant activation on the catalysts

**Fig. 4:**
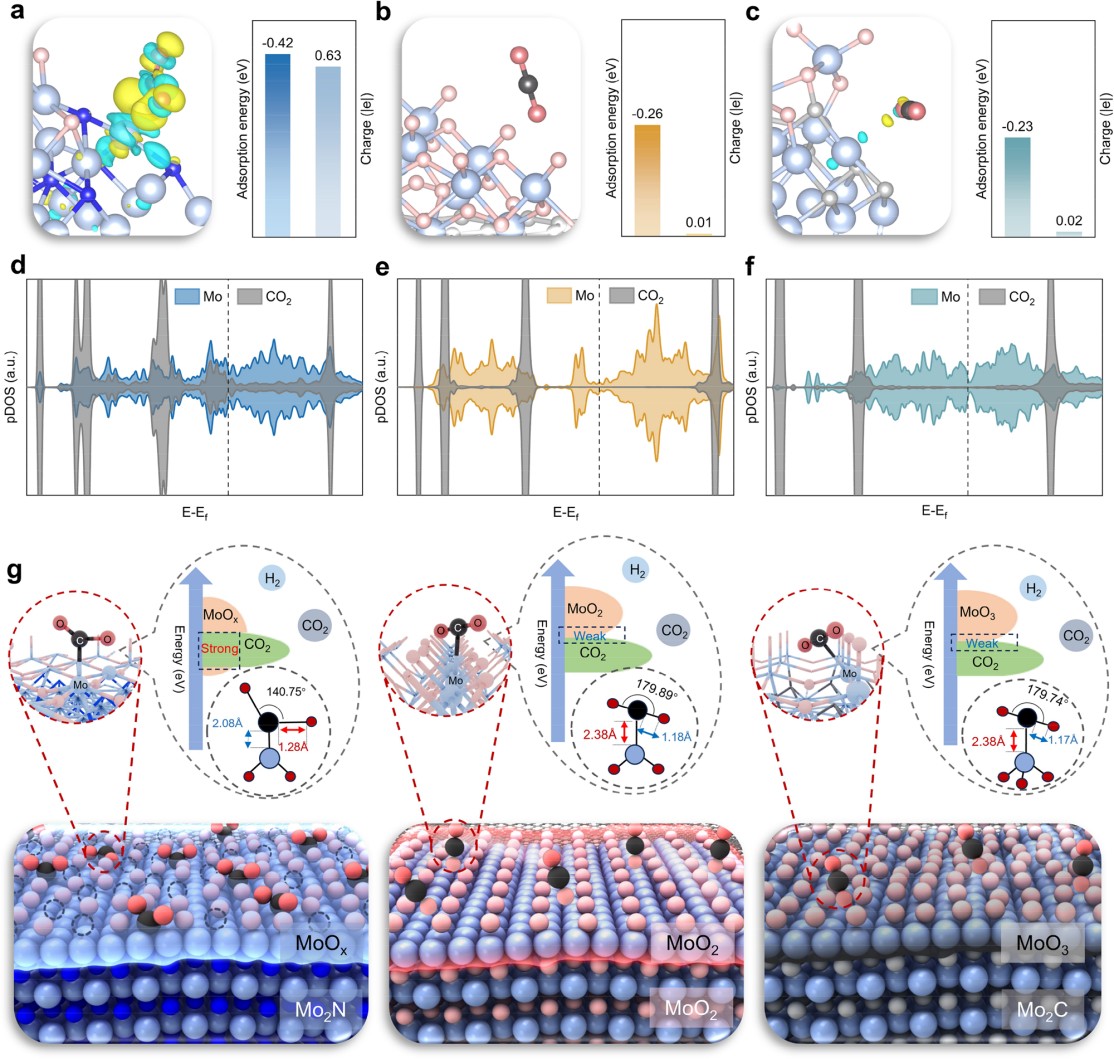
Surface reactant activation on the different heterostructures and metastable MoO_x_ site. Differential electron density plot, calculated adsorption energy and Bader charge of CO_2_* on **a** MoO_3_/Mo_2_N-C, **b** MoO_3_/MoO_2_-C, and **c** MoO_3_/Mo_2_C-C (cyan and yellow show charge consumption and accumulation, respectively, the cut-off of the density-difference iso-surface is 0.004 e·Bohr^−3^; color codes: light blue, molybdenum; blue, nitrogen; pink, oxygen in lattice; red, oxygen in molecule; gray, carbon in lattice; darkgray, carbon in molecule). PDOS analysis of CO_2_* on **d** MoO_3_/Mo_2_N-C, **e** MoO_3_/MoO_2_-C, and **f** MoO_3_/Mo_2_C-C. **g** Schematic illustration of the CO_2_* on the catalyst surfaces.

Molecular dynamics (MD) and DFT simulations were performed to investigate the CO_2_ adsorption properties on MoO_3_/Mo_2_N-C, MoO_3_/MoO_2_-C, and MoO_3_/Mo_2_C-C catalysts under RWGS reaction conditions (Supplementary Fig. 12). The distances distribution of CO_2_ molecules on the catalyst surface is statistically analyzed within 1.5 nm, where MoO_3_/Mo_2_N-C exhibits the highest proportion of gas molecules in close distance to the surface (around 4 Å) indicating the strongest CO_2_ adsorption capability on the MoO_3_/Mo_2_N-C surface (Supplementary Fig. 13). As shown in Fig. 4a–c and Supplementary Fig. 14a–c, the calculated adsorption energy demonstrates the strong binding ability of CO_2_ on MoO_3_/Mo_2_N-C (−0.42 eV) compared to MoO_3_/MoO_2_-C (−0.26 eV) and MoO_3_/Mo_2_C-C (−0.23 eV). Moreover, theoretical simulations indicate that the CO_2_ molecule exhibits obvious activation upon adsorption on the MoO_3_/Mo_2_N-C surface, which is reflected in the bending of molecular configuration, the elongation of chemical bonds, and the gain of electron density from Mo sites. The differential electron density analysis in Fig. 4a–c and Supplementary Fig. 14 show that the CO_2_ molecule adsorbed on MoO_3_/Mo_2_N-C owns the largest angular bending (from 180.00° to 140.75°), the longest C=O bond length (from 1.18 Å to 1.28 Å), and the maximum electron transfer (0.63 |e|), while the adsorption of CO_2_ is much weaker on both MoO_3_/MoO_2_-C and MoO_3_/Mo_2_C-C, on which the adsorbed CO_2_ molecule exhibits a linear configuration. PDOS plots (Fig. 4d–f and Supplementary Fig. 15) reveal that the electron state distribution changes significantly, with a substantial overlap of states between Mo and CO_2_ after the adsorption of CO_2_ on MoO_3_/Mo_2_N-C, further supporting the above results. Similarly, the adsorption and activation of H_2_ on MoO_3_/Mo_2_N-C exhibit the same results when compared with MoO_3_/MoO_2_-C and MoO_3_/Mo_2_C-C (Supplementary Figs. 16, 17). These calculations show that the unique Mo_2_N substrate structure endows the surface MoO_x_ center with enhanced CO_2_ and H_2_ activation capabilities, facilitating subsequent catalytic reactions. Figure 4g schematically summarizes the differences in CO_2_ activation for the three catalysts as a result of different surface reconstructions.

### CO_2_ hydrogenation performances and surface reconstructions in the RWGS reaction

**Fig. 5:**
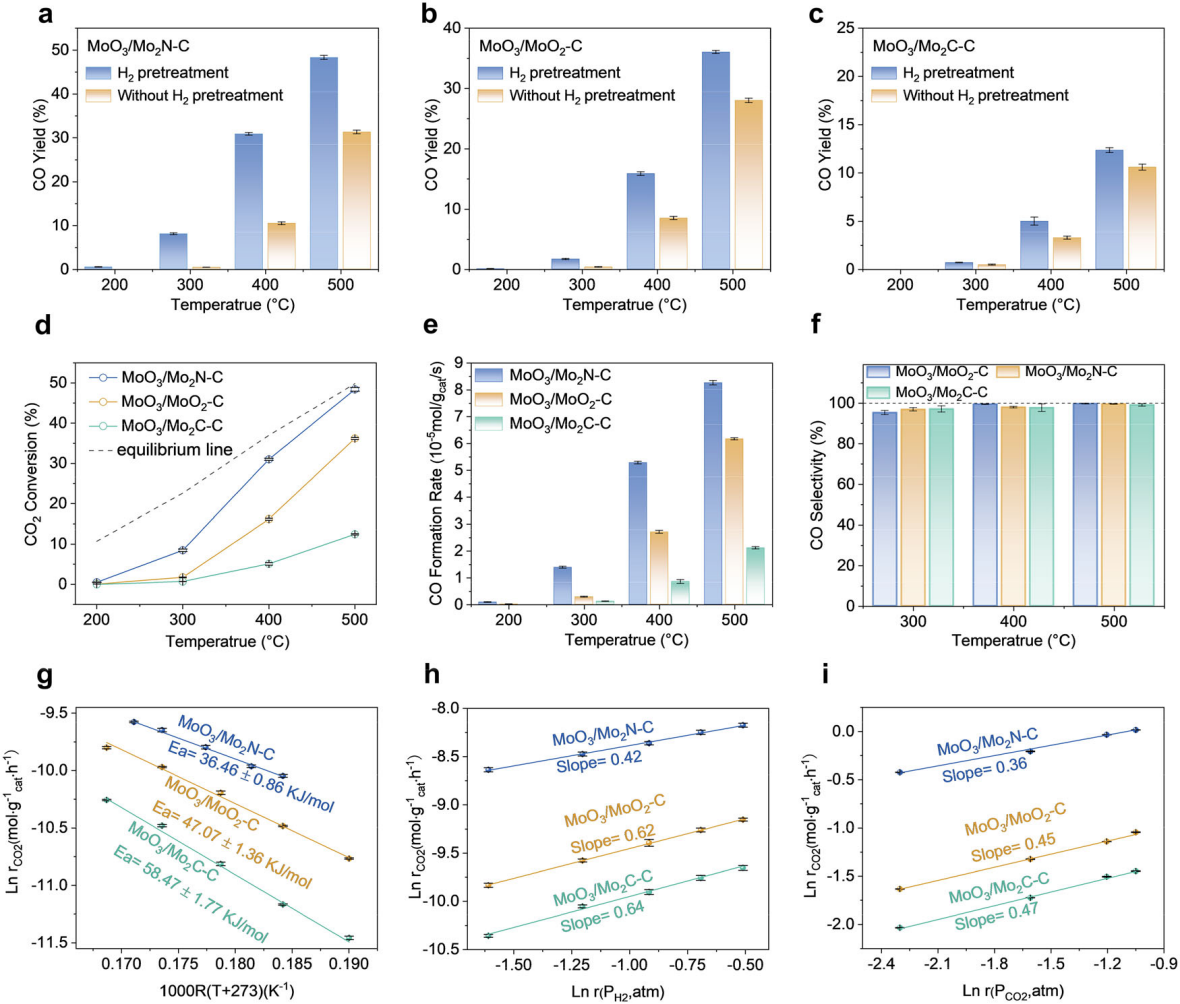
CO_2_ hydrogenation performances of molybdenum-based catalysts. **a**–**c** Catalytic results for CO yield under different temperatures, **d** CO_2_ conversion at various temperatures under 60,000 mL·g_cat_^−1^h^−1^, **e** Catalytic reaction rates, **f** CO selectivity, **g** Apparent activation energy (Ea), **h**, **i** Kinetic orders of reactants (H_2_ and CO_2_) for MoO_3_/Mo_2_N-C, MoO_3_/MoO_2_-C, and MoO_3_/Mo_2_C-C catalysts. (*n* = 3 independent experiments, data are presented as mean values ± SD).

We assessed the CO_2_ hydrogenation catalytic efficiency of the MoO_3_/Mo_2_N-C, MoO_3_/MoO_2_-C, and MoO_3_/Mo_2_C-C catalysts in the RWGS reaction, both before and after H_2_ pretreatment, within a fixed-bed reactor. The tests were conducted at a WHSV of 60,000 mL·g_cat_^−1^h^−1^. The MoO_3_/Mo_2_N-C catalyst shows an initial CO yield of 10.4 % at 400 °C, which increases significantly to 31.3% after H_2_ pretreatment, as depicted in Fig. 5a. At an elevated reaction temperature of 500 °C, the yield further rises to 48.3% and thus approaches the equilibrium conversion limit under the given WHSV. In contrast, the H_2_-pretreated MoO_3_/MoO_2_-C and MoO_3_/Mo_2_C-C catalysts yield considerably lower CO efficiencies of 15.8% and 4.9% at 400 °C and after H_2_ pretreatment, respectively (Fig. 5b, c). At 500 °C under the same WHSV, a CO_2_ conversion of 48.2%, 36.5%, 12.6% and CO formation rate of 8.26 × 10^−5^, 6.16 × 10^−5^, 2.11 × 10^−5^ mol_CO2_ g_cat_^‒1^ s^‒1^ is obtained for MoO_3_/Mo_2_N-C, MoO_3_/MoO_2_-C, MoO_3_/Mo_2_C-C catalysts, respectively (Fig. 5d, e). Notably, all catalysts exhibit exceptional CO selectivity exceeding 99 % across varying temperatures and space velocities. (Fig. 5f). In addition, the structure of the supports (Mo_2_N, MoO_2_, Mo_2_C) of the three catalysts do not change during the H_2_ treatment process (Supplementary Fig. 18). The apparent activation energy (Ea) of the MoO_3_/Mo_2_N-C catalyst is determined to be around 36 kJ mol^‒1^, which is significantly lower than that of MoO_3_/MoO_2_-C (47 kJ mol^−1^) and only two-thirds of that of MoO_3_/Mo_2_C-C (58 kJ mol^−1^). The resulting higher catalytic efficiency of MoO_3_/Mo_2_N-C is thus closely related to surface reconfiguration of MoO_3_ to MoO_x_ during the reaction (Fig. 5g). As shown in Fig. 5h, i, the reaction orders for H_2_ are 0.42, 0.62 and 0.64 and for CO_2_ 0.36, 0.45, and 0.47 for MoO_3_/Mo_2_N-C, MoO_3_/MoO_2_-C, MoO_3_/Mo_2_C-C, respectively. The higher reaction orders of MoO_3_/MoO_2_-C and MoO_3_/Mo_2_C-C for CO_2_ and H_2_ than for MoO_3_/Mo_2_N-C suggest that the adsorption and activation of reactant molecules are more hindered on their surfaces than for MoO_3_/Mo_2_N-C. On the other hand, the low CO_2_ reaction order on MoO_3_/Mo_2_N-C shows that the presence of MoO_x_ promotes the adsorption and activation of reactant molecules. We conducted stability tests on the best-performing catalysts. As shown in Supplementary Fig. 19, the MoO_3_/Mo_2_N-C catalyst exhibits excellent stability, maintaining more than 96.7% of its initial activity after the 200-hour evaluation.

To investigate the impact of surface molybdenum species reconstruction on activity in our catalysts, we controlled variables including particle size, specific surface area, and carbon content across the three catalysts. Since all catalysts were obtained from the same precursor via different calcination temperatures, their physical properties (e.g., particle size, surface area) are highly similar. As shown in Supplementary Figs. 20, 21, particle size distribution histograms and Brunauer-Emmett-Teller (BET) surface areas calculated from nitrogen adsorption-desorption isotherms confirm that MoO_3_/Mo_2_N-C, MoO_3_/MoO_2_-C, and MoO_3_/Mo_2_C-C exhibit comparable particle sizes (1.62 ± 0.49 nm, 1.68 ± 0.61 nm, and 1.99 ± 1.30 nm) and specific surface areas (2.60 m^2^·g^−1^, 2.38 m^2^·g^−1^, and 4.08 m^2^·g^−1^). Furthermore, XPS analysis (Supplementary Fig. 22) quantifies the carbon layer proportion in each catalyst. Due to carbon contributions from both the carbon layer and molybdenum carbide in MoO_3_/Mo_2_C-C, we included pure Mo_2_C XPS as a reference, estimating the carbon content within the carbon layer of MoO_3_/Mo_2_C-C. As shown in Supplementary Fig. 22b, the carbon content in pure Mo_2_C is ~44.4 at. %. Therefore, the carbon content in the carbon layer of the MoO_3_/Mo_2_C-C catalyst can be estimated at ~31.6 at.%, indicating that the carbon layer content is similar across all three catalysts.

To compare and understand the specific role of the oxide layer on the catalyst surface on CO_2_ hydrogenation, we synthesized Mo_2_N-C (500 °C, Ar, 2 h) and MoO_2_-C (700 °C, Ar, 2 h) in situ under identical reactor conditions, preventing surface MoO_3_ formation by avoiding air exposure, and immediately conducted RWGS reactions. Thermogravimetric analysis (TGA) determines precise feedstock loading to ensure consistent reaction conditions between bare supports and oxide-coated catalysts (Supplementary Fig. 23). Specifically, when the WHSV is 60,000 mL·gcat⁻¹h⁻¹ and the catalyst feed mass is 100 mg, the precursor mass loss at 500 °C is 79.7%. When preparing the Mo_2_N-C catalyst, the precursor feed mass is 125 mg. At 700 °C, the mass loss of the precursor is 56.1%, and the precursor feed mass is 178 mg when preparing the MoO_2_-C catalyst. Furthermore, XRD characterization of synthesized Mo_2_N-C and MoO_2_-C (Supplementary Fig. 24) shows unchanged diffraction peaks, confirming preserved lattice structures, indicating that Mo_2_N-C and MoO_2_-C differ from MoO_3_/Mo_2_N-C and MoO_3_/MoO_2_-C solely by lacking the oxide layer.

Supplementary Fig. 25 shows that the CO_2_ conversion rate and CO yield of the bare support Mo_2_N-C and MoO_2_-C without a surface MoO_3_ oxide layer are both lower than those of MoO_3_/Mo_2_N-C and MoO_3_/MoO_2_-C. After eliminating minor differences in feedstock quantity through the CO formation rate, it can be observed that the CO formation rate of Mo_2_N-C is reduced from 8.26 × 10^−5^ mol_CO2_ g_cat_^‒1^ s^‒1^ to 5.19 × 10^−5^ mol_CO2_ g_cat_^‒1^ s^‒1^ compared to MoO_3_/Mo_2_N-C, and the CO formation rate for MoO_2_-C is reduced from 6.17 × 10^−5^ mol_CO2_ g_cat_^‒1^ s^‒1^ to 4.80 × 10^−5^ mol_CO2_ g_cat_^‒1^ s^‒1^ compared to MoO_3_/MoO_2_-C. The performance of the bare support Mo_2_N-C decreases more significantly. Additionally, as shown in Supplementary Fig. 26, we have included measurements of activation energies and reaction orders for oxide-free Mo_2_N-C and MoO_2_-C. The apparent activation energy (E_a_) of the Mo_2_N-C catalyst is determined as 47.96 kJ·mol⁻¹, significantly lower than that of MoO_2_-C (65.00 kJ·mol⁻¹). Concurrently, the H₂ reaction order for Mo_2_N-C (0.36) is markedly lower than for MoO_2_-C (0.61), following the same trend observed for the MoO_3_/Mo_2_N-C and MoO_3_/MoO_2_-C catalysts. However, Mo_2_N-C and MoO_2_-C exhibit similar CO_2_ reaction orders (0.64 and 0.71), revealing a distinct pattern. This indicates that the surface MoO_3_ layer has minimal impact on the H_2_ reaction order but significantly modulates the CO_2_ reaction order.

To clarify the role of the oxide layer, we compared CO_2_ and H_2_ reaction orders and activation energies between catalysts with and without surface oxides. As shown in Supplementary Fig. 27, the CO_2_ reaction order for MoO_3_/Mo_2_N-C is 0.36, while the Mo_2_N-C exhibits an increased CO_2_ reaction order of 0.64; similarly, MoO_3_/MoO_2_-C shows a CO_2_ reaction order of 0.45 versus MoO_2_-C at 0.71. The lower CO_2_ reaction orders signify superior CO_2_ activation capability in MoO_3_/Mo_2_N-C and MoO_3_/MoO_2_-C catalysts, which originates directly from the surface MoO_3_ layer. Concurrently, the negligible difference in H_2_ reaction orders confirms that H_2_ activation capability is independent of the oxide layer. Notably, oxide-free catalysts exhibit higher activation energies (Supplementary Fig. 28), likely due to impaired CO_2_ adsorption and activation without surface MoO_3_, resulting in higher temperature dependency.

To elucidate the catalytic nature of MoO_3_/Mo_2_N-C, we comprehensively compared the RWGS performance metrics of the MoO_3_/Mo_2_N-C catalyst, the passivated MoO_3_/Mo_2_N-C catalyst (lacking MoO_x_), and the Mo_2_N-C catalyst (lacking the surface MoO_3_ oxide layer). As shown in Supplementary Fig. 29a–e, the MoO_3_/Mo_2_N-C catalyst exhibits the highest CO_2_ conversion and CO formation rate, lowest CO_2_ reaction order and activation energy. These results suggest that MoO_x_, rather than MoO_3_, serves as the primary active species in the catalytic system. In contrast, the Mo_2_N-C catalyst demonstrates the lowest H_2_ reaction order, indicating the strongest H_2_ activation capability when Mo_2_N is fully exposed. The CO_2_ and H_2_ reaction orders, as well as the activation energy, reflect the dependence of the catalytic reaction on CO_2_ concentration, H_2_ concentration, and temperature, respectively. Supplementary Fig. 29f visually summarizes the roles of Mo_2_N and MoO_x_ in the RWGS reaction mechanism, demonstrating that the exposure of Mo_2_N reduces the reaction’s dependence on H_2_ concentration, while MoO_x_ significantly reduces its dependence on both CO_2_ concentration and temperature.

Additionally, we synthesized unsupported Mo_2_N and Mo_2_C via ammonia and methane reduction methods (SEM, Supplementary Fig. 30), with XRD characterization confirming perfect alignment of all lattice diffraction peaks with standard Mo_2_N and Mo_2_C reference patterns. Subsequent Raman analysis reveals oxide layers on the unsupported Mo_2_N and Mo_2_C surfaces, where Mo_2_N exhibits stronger MoO_3_ Raman signals than Mo_2_C, which is consistent with trends observed for MoO_3_/Mo_2_N-C and MoO_3_/MoO_2_-C catalysts (Supplementary Fig. 31). Furthermore, CO_2_ conversion tests in RWGS reactions before and after H_2_ treatment demonstrate unchanged performance for Mo_2_C but enhanced conversion rates for Mo_2_N, unequivocally establishing identical surface reconstruction behavior to our catalysts (Supplementary Fig. 32).

**Fig. 6:**
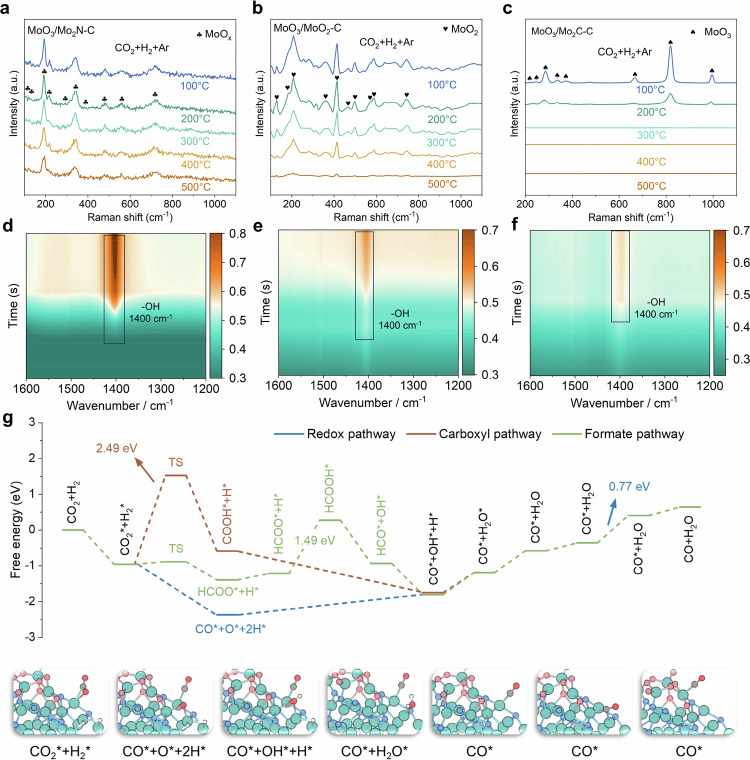
Mechanism exploration of molybdenum-based catalysts under the RWGS reaction. **a**–**c** In-situ Raman spectroscopy results of MoO_3_/Mo_2_N-C, MoO_3_/MoO_2_-C, and MoO_3_/Mo_2_C-C under RWGS reaction conditions. **d**–**f** In-situ DRIFT spectra of the RWGS reaction over MoO_3_/Mo_2_N-C, MoO_3_/MoO_2_-C, and MoO_3_/Mo_2_C-C at 25–500 °C (Pretreatment condition: 500 °C in H_2_ diluted in Ar stream (1 mL min^−1^ for H_2_ and 9 mL min^−1^ for Ar) for 1 h. Reaction conditions: 12 % H_2_ and 4 % CO_2_ in Ar (H_2_/CO_2_ molar ratio of 3) at 10 mL min^−1^. **g** Free energy profiles of the three reaction pathways (redox, carboxyl, and formate) on MoO_3_/Mo_2_N-C. The configurations of intermediates in the redox route are displayed at the bottom, while those in the other two pathways (carboxyl and formate) are shown in Supplementary Fig. 38.

To get further insight into the dynamic surface transformations of the catalysts throughout the RWGS reaction, in situ Raman spectroscopy was employed over a temperature range of 100–500 °C, as shown in Fig. 6a–c. As illustrated in Fig. 6a for MoO_3_/Mo_2_N-C, the surface MoO_3_ is reduced already at 100 °C, as only peaks corresponding to MoO_x_ are detectable across the entire temperature range. The in-situ formation of MoO_x_ species introduces many oxygen vacancies, which are postulated to be the predominant active sites catalyzing the RWGS reaction. For the MoO_3_/MoO_2_-C catalyst, the Raman spectra depicted in Fig. 6b indicate that the surface MoO_3_ structure has already been reduced to MoO_2_ at 100 °C. The characteristic peaks attributed to MoO_2_ diminish with further temperature increase to 500 °C, suggesting that the surface MoO_2_ is likely to constitute the principal active phase in the MoO_3_/MoO_2_-C catalyst. Meanwhile, surface MoO_3_ in the MoO_3_/Mo_2_C-C catalyst demonstrates remarkable stability throughout the RWGS reaction from 100 to 500 °C, (Fig. 6c). Note that due to the small amount of MoO_3_ on the surface and the increasing blackbody radiation with temperature, the peaks of MoO_3_ seem to disappear at ~300 °C, however no peaks of MoO_x_ or MoO_2_ can be identified instead. To further support our findings, commercial MoO_3_ was examined under identical conditions. Supplementary Fig. 33 reveals that the MoO_3_ surface remains unaltered even at the elevated temperature of 500 °C during the RWGS reaction, with no signals of MoO_2_ or MoO_x_. These observations suggest that the reduction of MoO_3_ is confined to the heterostructured surface, with the nature of the reduced species closely linked to the specific substrate. The MoO_3_/Mo_2_N heterostructure proves to be most suitable for the formation of a highly active MoO_x_ surface, resulting in the superior performance of the MoO_3_/Mo_2_N-C catalyst. This insight into the substrate-dependent reducibility and surface stability of MoO_3_-based catalysts provides a compelling rationale for the observed variations in catalytic activity and selectivity in the RWGS reaction.

Building upon our investigation, in-situ diffuse reflectance infrared Fourier transform spectroscopy (DRIFTS) was employed to discern the intermediate species present on the MoO_3_/MoO_2_-C, MoO_3_/Mo_2_N-C, and MoO_3_/Mo_2_C-C catalysts throughout the RWGS reaction within a temperature range of 50–500 °C. The DRIFTS spectra, as depicted in Fig. 6d–f and Supplementary Figs. 34–36, exhibit a prominent peak at 1400 cm^−1^, which is attributed to the vibration of hydroxyl groups generated from the activation of H_2_ on the catalyst surfaces^36,37^. Moreover, the hydroxyl vibrational peak at 1400 cm^-1^ is present throughout the entire RWGS reaction process for all three catalysts. This observation is essential, as it suggests that the MoO_3_/MoO_2_-C, MoO_3_/Mo_2_N-C, and MoO_3_/Mo_2_C-C catalysts predominantly adhere to a redox mechanism, diverging from the traditional associative mechanism. In the redox mechanism, CO_2_ is adsorbed onto the catalyst surface, where it undergoes direct C=O bond cleavage under the catalyst’s influence to yield CO and an oxygen species O* adsorbed on the catalyst surface. The O* is then reduced by H_2_ to form H_2_O, which, after desorption, initiates a new cycle. Consequently, this alternative pathway circumvents the formation of substantial bicarbonate intermediates, thereby culminating in exceptional selectivity for the RWGS reaction. The DRIFTS findings, in conjunction with the Raman spectroscopy results, corroborate the substrate-dependent reducibility and active site formation on the MoO_3_/Mo_2_N-C catalyst, which is crucial for its superior catalytic performance. Isotope labeling experiments have been employed to trace the origin of reactant components. We utilized a feed gas mixture of 2.5% ^13^CO_2_ + 97.5% Ar and employed in situ mass spectrometry to distinguish the carbon source of the CO product. As shown in Supplementary Fig. 37, when ^13^CO_2_ is pulsed over the MoO_3_/Mo_2_C-C catalyst in the absence of H_2_, the C=O bond in ^13^CO_2_ cleave, generating ^13^CO; no ^12^CO is observed. This confirms that neither the surface MoO_3_ layer nor the bulk Mo_2_C in the MoO_3_/Mo_2_C-C catalyst underwent a carbon exchange mechanism during the RWGS reaction. Consequently, all carbon in the produced CO originates exclusively from CO_2_.

Using first-principles calculations, we further explored the catalytic reaction mechanism from an atomic-scale perspective. Here, both the redox and the associative (including the carboxyl and the formate pathways) mechanisms were considered (Fig. 6g and Supplementary Fig. 38). For the associative mechanism, the hydrogenation of CO_2_ on MoO_3_/Mo_2_N-C is more hindered, especially for the carboxyl pathway with a significantly higher kinetic energy barrier of 2.49 eV, which limits the subsequent reaction. For the formate pathway, the formation of HCOOH* intermediate is hindered due to a higher thermodynamic energy barrier of 1.49 eV. Regarding the redox mechanism, computational simulations reveal that CO_2_* tends to form CO* and O* spontaneously without any kinetic barrier, and the subsequent reaction process shows mild energy changes with a much lower rate-determining step energy barrier of only 0.77 eV. Thus, compared to the associative mechanism, the RWGS reaction is more likely to follow the redox pathway on MoO_x_ formed on the Mo_2_N surface, aligning with experimental results. Furthermore, through a detailed analysis of the redox pathways, we systematically investigated the transformation and desorption pathways of key intermediates (O, OH, and CO). As illustrated in Supplementary Fig. 39, CO_2_ and H_2_ undergo catalytic activation on the surface to form adsorbed intermediates: CO*, O*, and H*. The reaction proceeds via the sequential hydrogenation of the O* intermediate, first forming OH*, followed by H_2_O*, which ultimately desorbs. The remaining CO* intermediate subsequently desorbs, completing the catalytic cycle and regenerating the active surface. Notably, a comparative analysis of potential pathways reveals that this mechanism possesses the most favorable thermodynamics, with an energy barrier of only 0.77 eV, significantly lower than those of other pathways (0.97, 0.97, and 1.16 eV).

## Discussion

In this study, we address the complex phenomenon of the surface sensitivity and dynamic nature of catalyst surface remodeling under high temperatures, which is essential for understanding and finally enhancing the catalytic efficiency of heterogeneous catalysts. While the interactions between active materials and substrates in supported catalyst systems have been extensively studied, the impact of substrate type on surface reconstruction in Mo-based catalysts has remained largely unexplored. Our work sheds new light on the intricate chemistry of catalyst surface reconstruction and its dependence on substrate type, revealing a direct correlation between the reducibility of surface MoO_3_ and the nature of the underlying Mo-based materials (Mo_2_N, MoO_2_, and Mo_2_C). Comprehensive in-situ characterization techniques and theoretical analyses reveal that the MoO_3_ layer on MoO_2_ and Mo_2_N is in-situ reduced to MoO_2_ or metastable MoO_x_ (2 < x < 3), respectively, but not reduced at all on Mo_2_C, during the catalysis process. We demonstrate that the surface stress exerted by different substrates on MoO_3_ leads to different oxidation states, with the MoO_3_/Mo_2_N-C catalyst exhibiting an optimal level of reducibility that stabilizes metastable MoO_x_ active sites, which shows an unprecedented CO yield of up to 48.2%, approaching the equilibrium conversion limit, the CO formation rate of 8.23 × 10^−5^ mol_CO_ g_cat_^‒1^ s^‒1^, and up to 99% CO selectivity under 500 °C. These metastable species are crucial for the efficient activation of CO_2_; meanwhile, Mo_2_N, which possesses noble metal-like properties in H_2_ dissociation, facilitates a direct redox pathway on the MoO_3_/Mo_2_N-C catalyst. This pathway is responsible for the exceptional selectivity and stability observed in high-temperature RWGS reactions. Our findings not only deepen the understanding of how heterostructure interfaces influence catalytic performance but also offer valuable guidelines for developing efficient heterogeneous catalysts tailored for various catalytic reactions. By elucidating the interplay between substrate type and surface reconstruction, we pave the way for the design of catalysts with targeted properties, poised to unlock new possibilities in catalysis.

## Methods

### Synthesis of the organic-polyoxometalate cMoO_3_/crystals

Ammonium molybdate tetrahydrate ((NH_4_)_6_Mo_7_O_24_·4H_2_O) served as the Mo–POM precursor, with p-phenylenediamine as the organic ligand in an aqueous medium. In a typical procedure, 1.08 g of p-phenylenediamine was dissolved in 50 mL of deionized water, followed by the dropwise addition of 2.48 g of (NH_4_)_6_Mo_7_O_24_·4H_2_O dissolved in another 50 mL of water. Subsequently, 34 mL of 1 M HCl was introduced to modulate the morphology of the assembled co-crystals. The mixture was reacted for 3 h, after which the solid product was collected via filtration and thoroughly washed with water and ethanol.

### Synthesis of molybdenum-based catalysts

The as-synthesized precursors were carbonized under an Ar atmosphere at different temperatures—500 °C, 700 °C, and 900 °C—using a heating rate of 5 °C·min^−1^ and a 2 h dwell time. After cooling to room temperature, the resulting materials were passivated in a 1% O_2_/Ar flow for 12 h to form a protective surface MoO_3_ layer while avoiding bulk oxidation of the Mo₂N and Mo₂C phases. The final catalysts are denoted as MoO_3_/MoO_2_-C (500 °C), MoO_3_/Mo_2_N-C (700 °C), and MoO_3_/Mo_2_C-C (900 °C). Unless otherwise specified, these designations refer to samples carbonized at the respective temperatures.

### Catalytic measurements

The CO_2_ hydrogenation activity for the RWGS reaction was assessed in a fixed-bed reactor operating at atmospheric pressure. In each test, 100 mg of catalyst (sieve fraction) was diluted with 100 mg of inert SiO_2_ and loaded into a quartz tube. Prior to reaction, the catalyst was pretreated in a 15% H_2_/Ar stream (100 mL·min^−1^) at 500 °C for 1 h. After cooling to room temperature, the feed was switched to the RWGS reaction mixture (23% CO_2_, 69% H_2_, 8% Ar) at a total flow rate of 100 mL·min^−1^. The reaction was maintained for 60 min at each temperature to reach steady state before product analysis. The system pressure was maintained at 1 bar using a back-pressure regulator. Effluent gases were analyzed online using an Agilent 7890B gas chromatograph equipped with two TCDs and one FID. Argon served as the internal standard for gas flow quantification. CO_2_ conversion was calculated based on the following expression:

1 $${X}_{{{CO}}_{2}}\left(\%\right)=\frac{{n}_{{in}}^{{{CO}}_{2}}-{n}_{{out}}^{{{CO}}_{2}}}{{n}_{{in}}^{{CO}2}} \times 100\%=\left(1-\frac{\frac{{A}_{{out}}^{{{CO}}_{2}}}{{A}_{{out}}^{{Ar}}}}{\frac{{A}_{{in}}^{{{CO}}_{2}}}{{A}_{{in}}^{{Ar}}}}\right)\times 100\%$$

where ${n}_{{in}}^{{{CO}}_{2}}$ is the concentration of CO_2_ in the reaction stream, and ${n}_{{out}}^{{{CO}}_{2}}$ is the concentration of CO_2_ in the outlet gas. ${A}_{{in}}^{{{CO}}_{2}}$ and ${A}_{{in}}^{{Ar}}$ refer to the chromatographic peak area of CO_2_ and Ar in the inlet gas, respectively, and ${A}_{{out}}^{{{CO}}_{2}}$ and ${A}_{{out}}^{{Ar}}$ refer to the chromatographic peak area of CO_2_ and Ar in the outlet gas, respectively. The chromatographic peak area of each component is proportional to the concentration of each component.

CO selectivity was calculated by the following equations:

2 $${{{{\rm{S}}}}}_{{{{\rm{CO}}}}}(\%)=\frac{\,{n}_{{out}}^{{CO}}}{{n}_{{out}}^{{CO}}+{n}_{{out}}^{{{CH}}_{4}}}\times 100\%=\frac{{A}_{{out}}^{{CO}} \times \,{f}_{{{{\rm{CO}}}}/{{{\rm{Ar}}}}}}{{A}_{{out}}^{{{CH}}_{4}}\times {f}_{{{CH}}_{4}/{{{\rm{Ar}}}}}+{A}_{{out}}^{{CO}}\times {f}_{{{{\rm{CO}}}}/{{{\rm{Ar}}}}}}\times 100\%$$

where ${n}_{{in}}^{{CO}}$ and ${n}_{{in}}^{{{CH}}_{4}}$ refer to the concentration of CO and CH_4_ in the outlet gas, respectively. ${f}_{{{{\rm{CO}}}}/{{{\rm{Ar}}}}}$ and ${f}_{{{{\rm{CH}}}}4/{{{\rm{Ar}}}}}$ are relative correction factors of CO to Ar and CH_4_ to Ar, respectively, which are determined by the calibrating gas. ${A}_{{out}}^{{CO}}$ and ${A}_{{out}}^{{{CH}}_{4}}$ are the chromatographic peak areas of CO and CH_4_ detected by the FID in the outlet gas.

The carbon balance was calculated as:

3 $${{{{\rm{C}}}}}_{{{{\rm{balance}}}}}(\%)=\frac{{n}_{{out}}^{{{CO}}_{2}}+{n}_{{out}}^{{CO}}+{n}_{{out}}^{{{CH}}_{4}}}{{n}_{{in}}^{{CO}}}\times 100\%$$

where ${n}_{{in}}^{{CO}}$ is the concentration of CO in the reaction stream, ${n}_{{out}}^{{{CO}}_{2}}$, ${n}_{{out}}^{{{CO}}_{2}}$, and ${n}_{{out}}^{{{CO}}_{2}}$ are the concentration of CO_2_, CO, and CH_4_ in the outlet gas.

## Supplementary information

Supplementary Information

Transparent Peer Review file

## Source data

Source Data

## Data Availability

All data supporting the findings of this study are available within this article and Supplementary Information or from the corresponding author upon request. The data generated in this study are provided in the Supplementary Information/Source Data file. Source data are provided with this paper.
