# Supplementary material for: Unraveling surface sensitivity for generating metastable active sites in molybdenum-based catalysts for CO2 hydrogenation
Source: Nat Commun. 2025 Nov 24;16:11323. doi: 10.1038/s41467-025-66430-3 (PMC12722216; doi:10.1038/s41467-025-66430-3)
Supplement: Supplementary file 1 — Supplementary Information [file 41467_2025_66430_MOESM1_ESM.pdf]

Supplementary Information for

Unraveling surface sensitivity for generating metastable active sites in molybdenum-based catalysts for CO_2 hydrogenation

This PDF file includes:

Supplementary Figures

Supplementary Methods

Supplementary References

Supplementary Figures

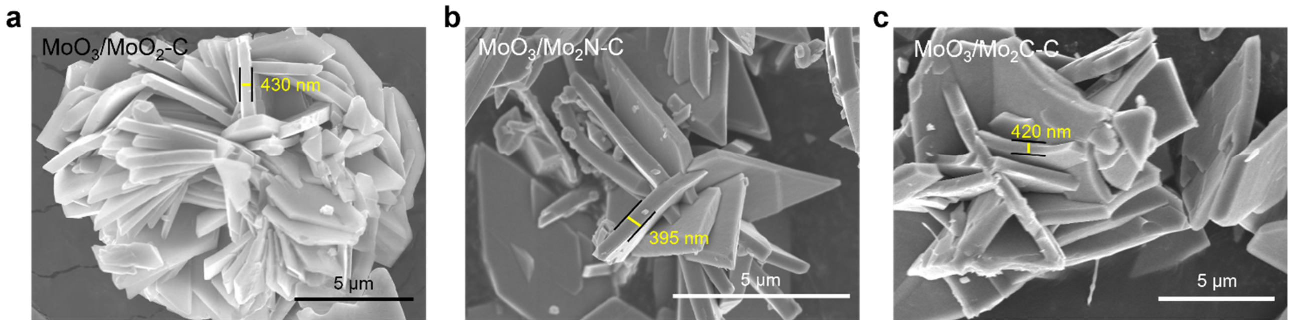

Supplementary Figure 1. SEM images of **a)** MoO_3/Mo_2N-C, **b)** MoO_3/MoO_2-C, **c)** MoO_3/Mo_2C-C structures.

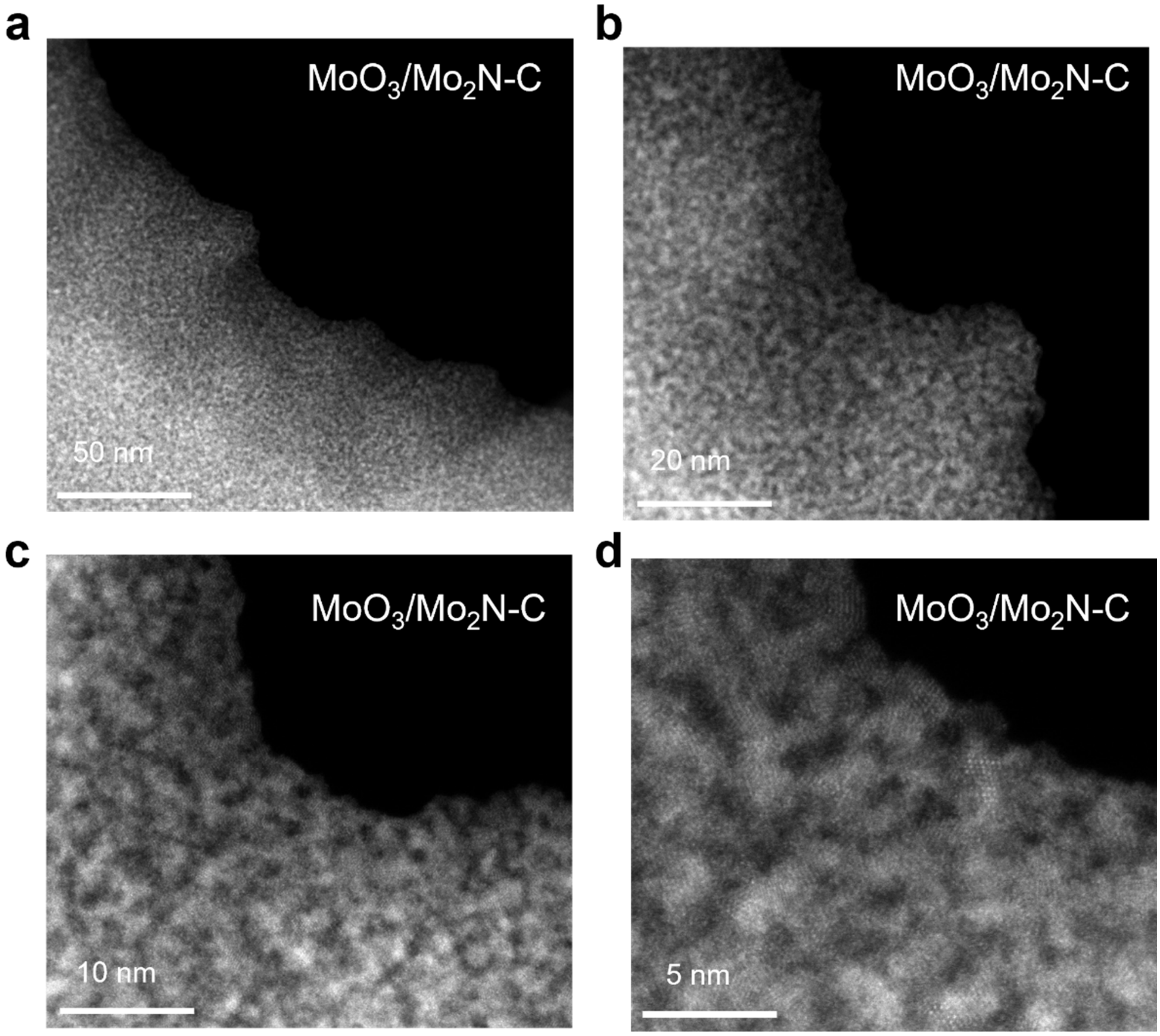

Supplementary Figure 2. HAADF-STEM images of the MoO_3/Mo_2N-C catalyst.

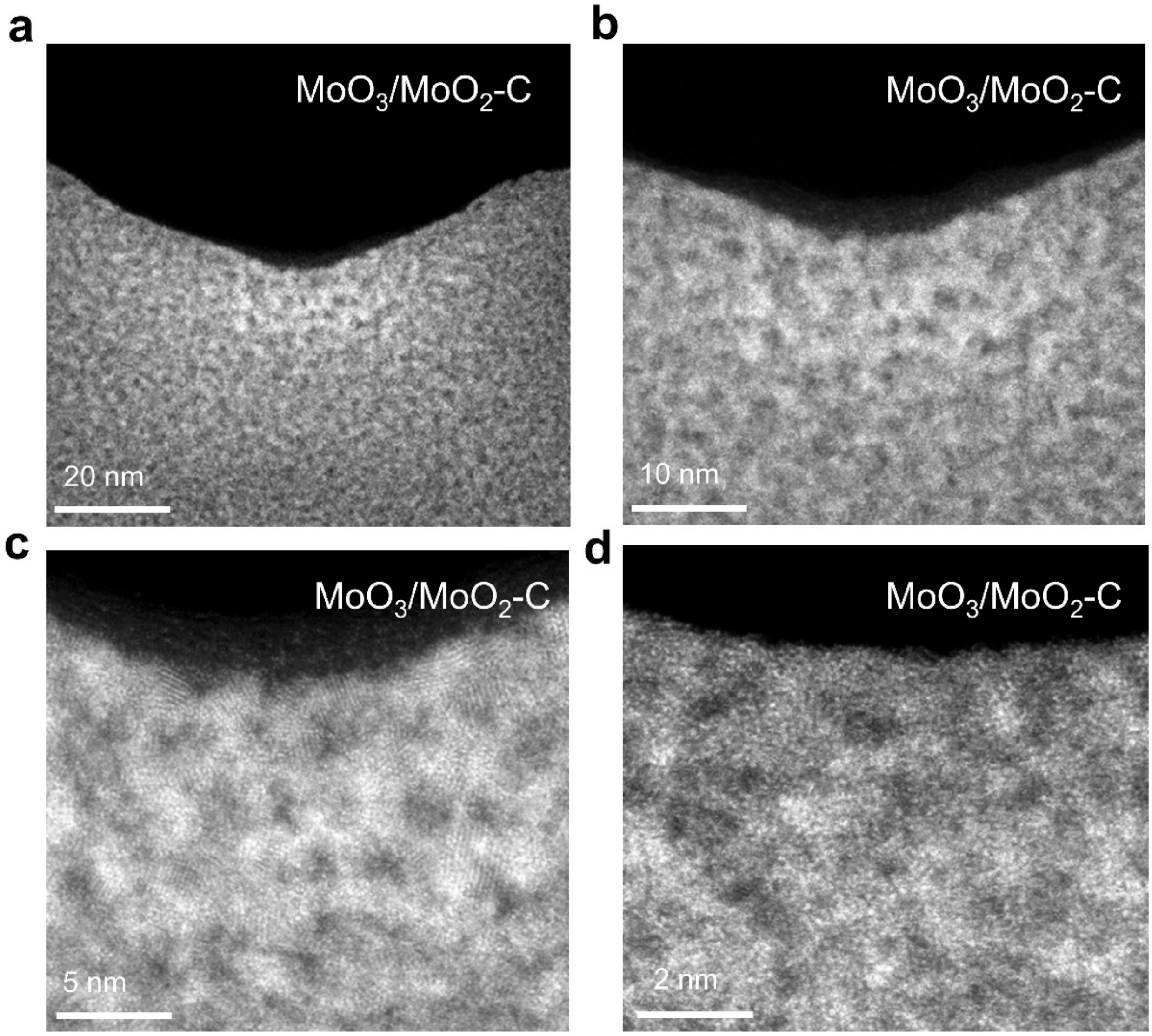

Supplementary Figure 3. HAADF-STEM images of the MoO_3/MoO_2-C catalyst.

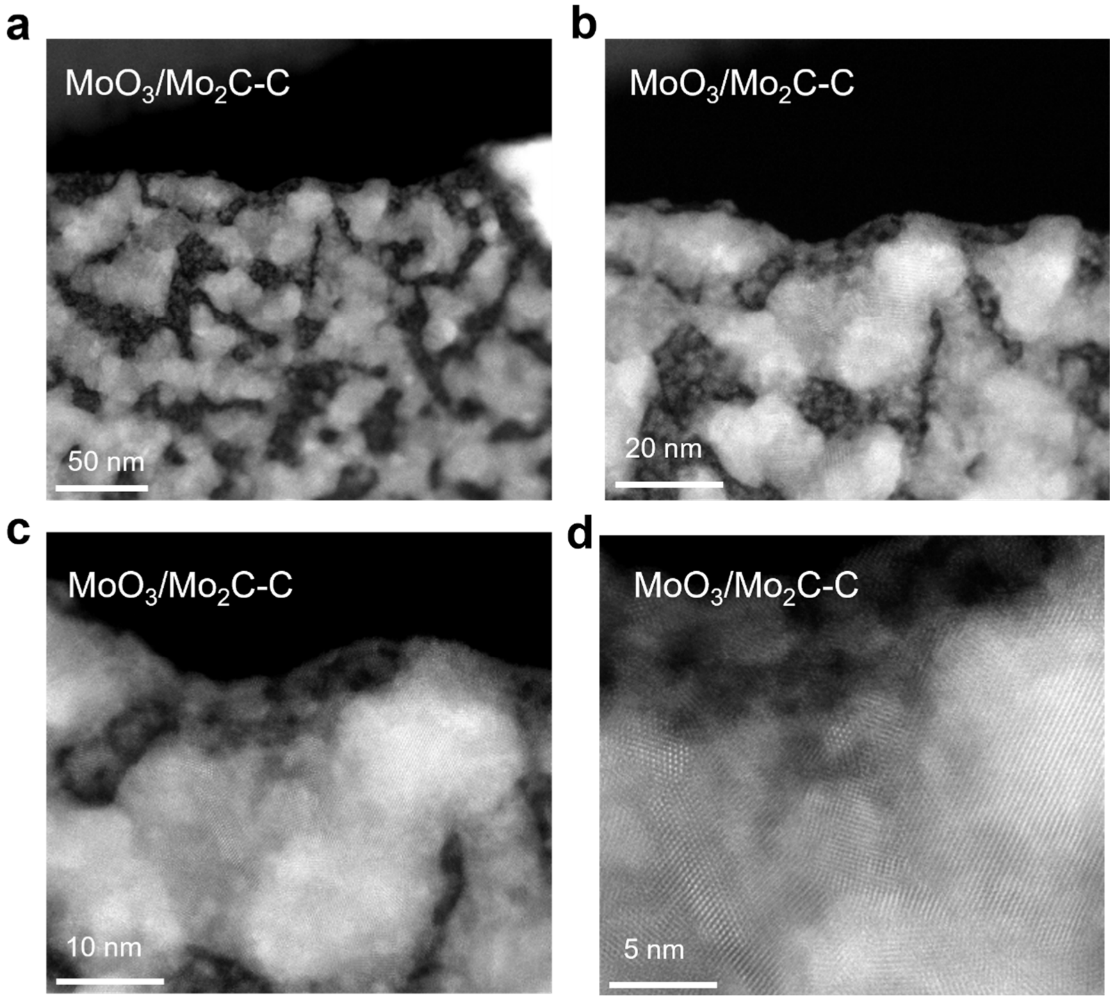

Supplementary Figure 4. HAADF-STEM images of the MoO_3/Mo_2C-C catalyst.

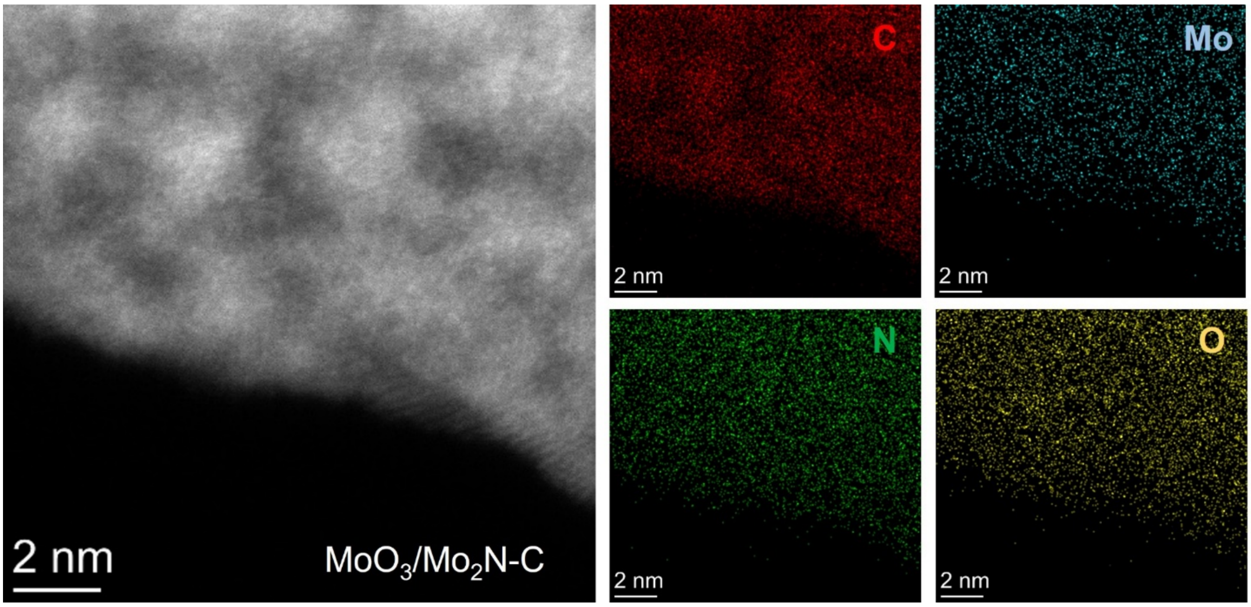

Supplementary Figure 5. STEM-EDS elemental mapping images of MoO_3/Mo_2N-C.

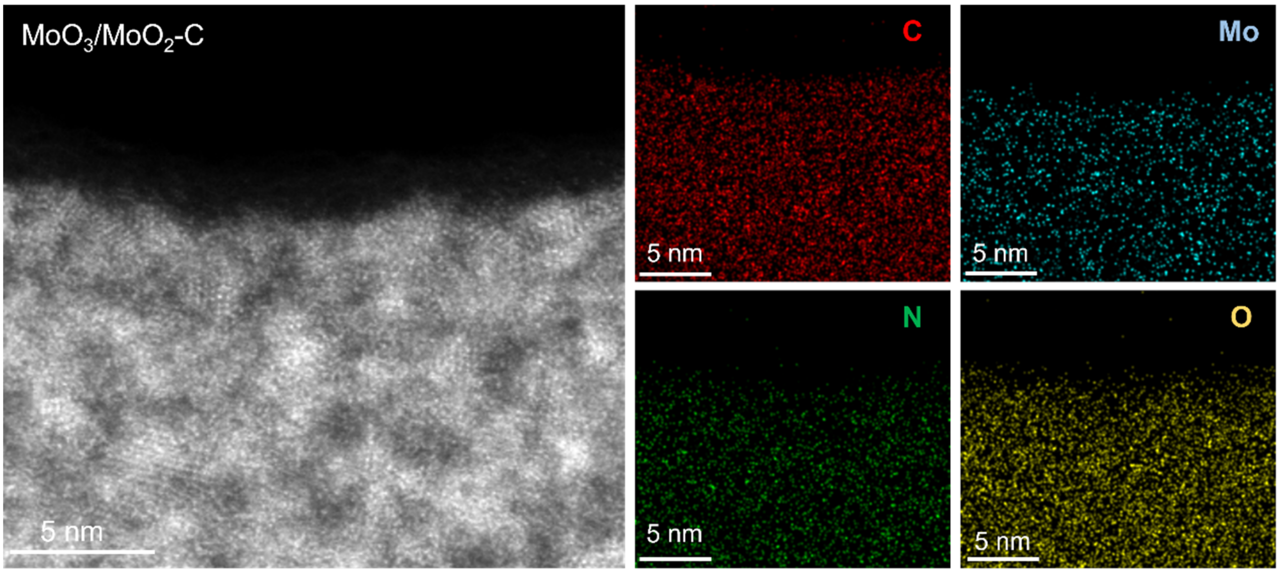

Supplementary Figure 6. STEM-EDS elemental mapping images of MoO_3/MoO_2-C.

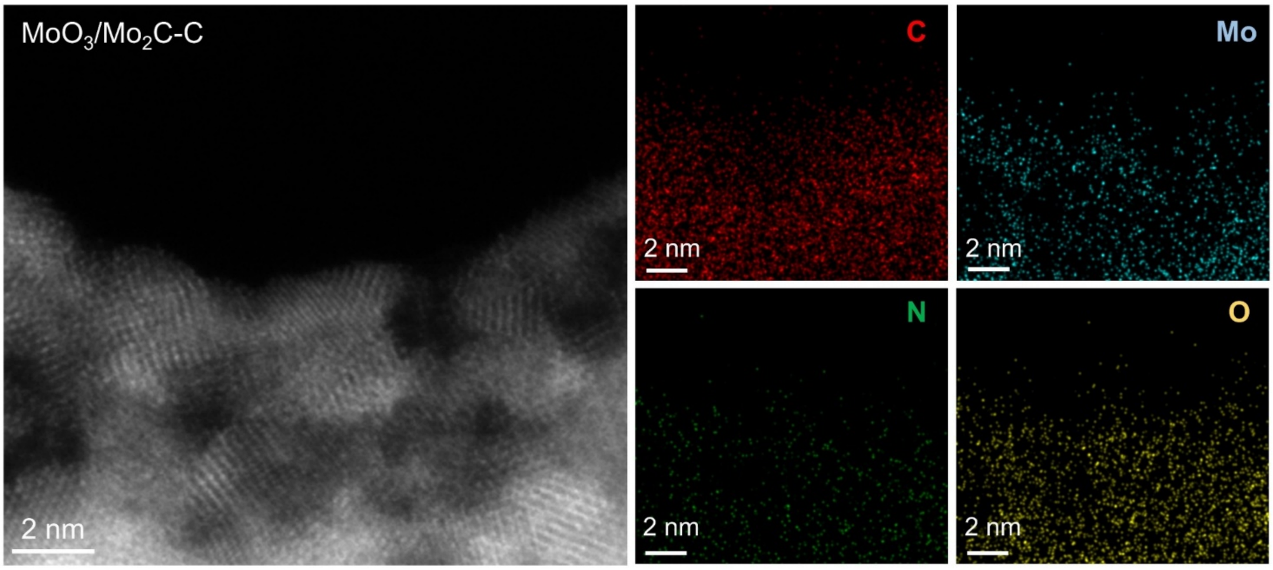

Supplementary Figure 7. STEM-EDS elemental mapping images of MoO_3/Mo_2C-C.

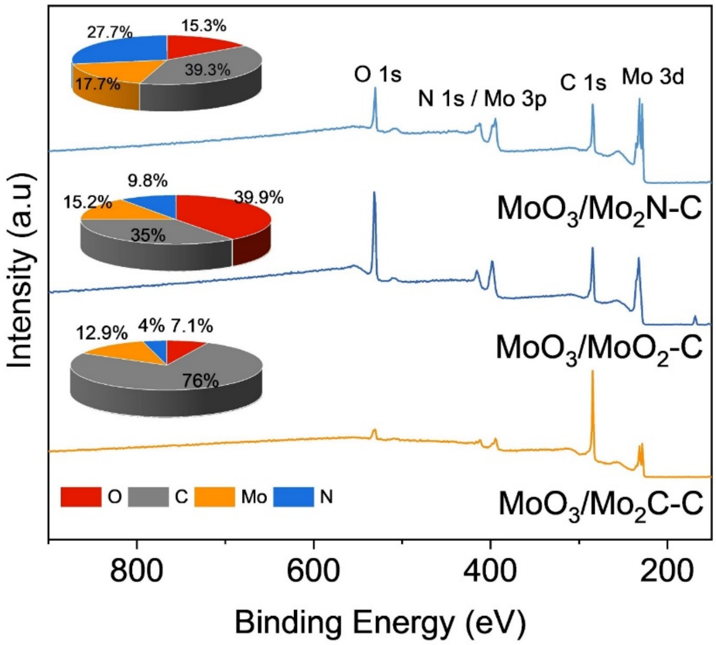

Supplementary Figure 8. XPS survey spectra and elemental content for MoO_3/Mo_2N-C, MoO_3/MoO_2-C, and MoO_3/Mo_2C-C.

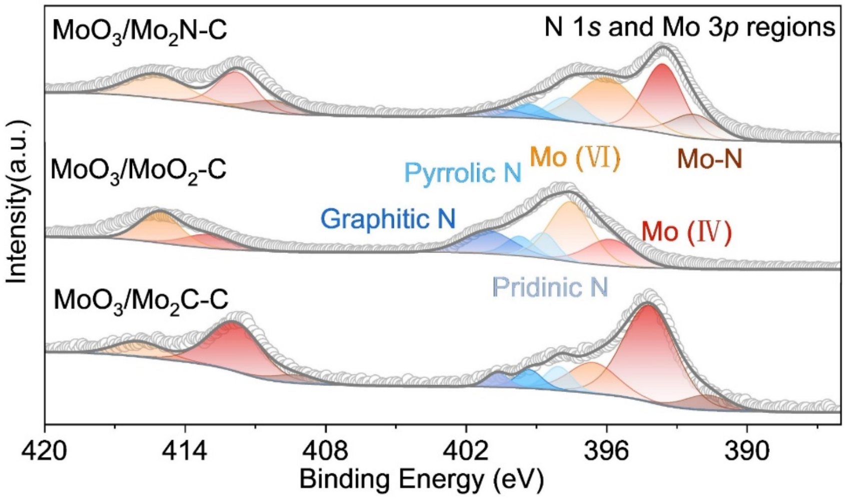

Supplementary Figure 9. High-resolution XPS peaks for MoO_3/Mo_2N-C, MoO_3/MoO_2-C, and MoO_3/Mo_2C-C catalysts of Mo 3*p* and N 1*s*.

Since the atomic orbitals binding energy of N and Mo are close, it should be noticed that there is a Mo 3*p* peak (394 eV) in the N 1*s* peak (394.9 eV) range. The N 1*s* region scan showed four obvious peaks at ~ 394.9, ~ 397.3, ~ 398.7, and ~ 401.0 eV, corresponding to Mo-N, pyridine N, pyrrole N, and graphite N, separately, where the highest Mo-N content was demonstrated in MoO_3/Mo_2N-C,

confirming the formation of Mo_2N species.

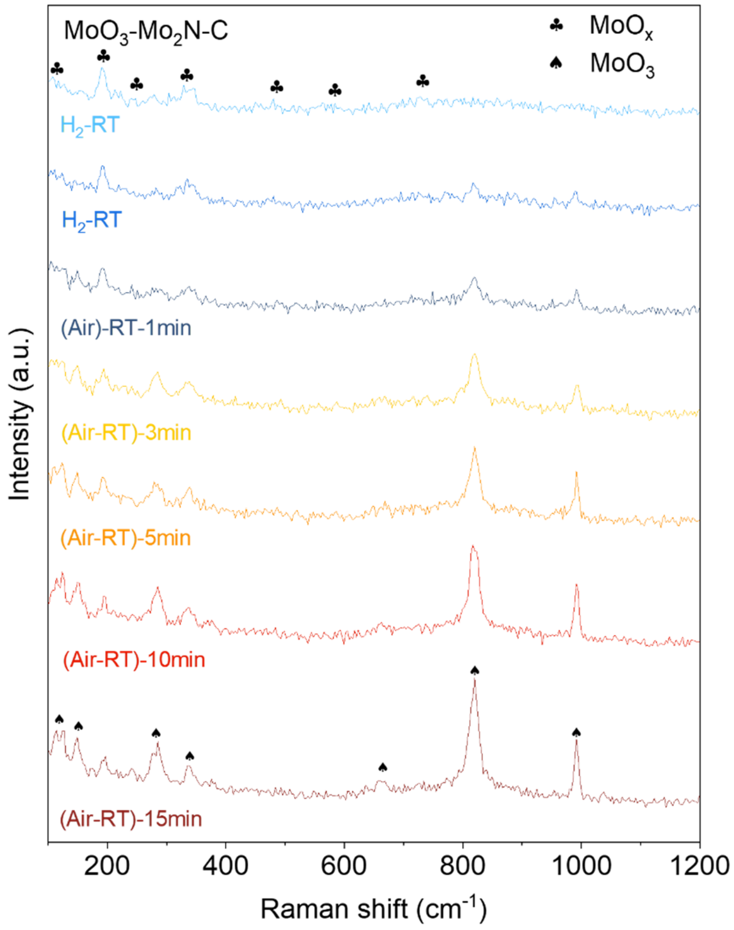

Supplementary Figure 10. In-situ Raman results of MoO_3/Mo_2N-C when the atmosphere is switched from H_2 to Air.

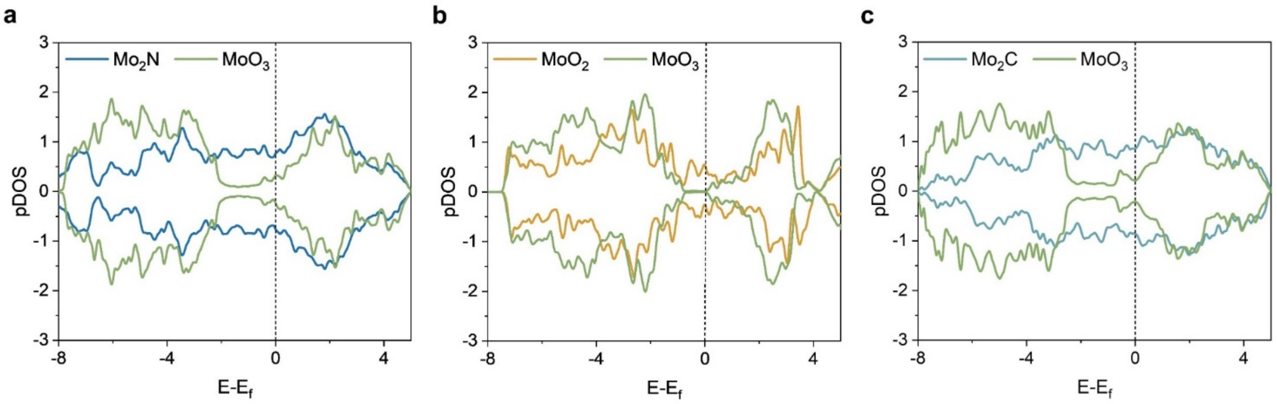

Supplementary Figure 11. PDOS analysis between substrate and surface oxide layer of **a)** MoO_3/Mo_2N-C, **b)** MoO_3/MoO_2-C, and **c)** MoO_3/Mo_2C-C catalysts.

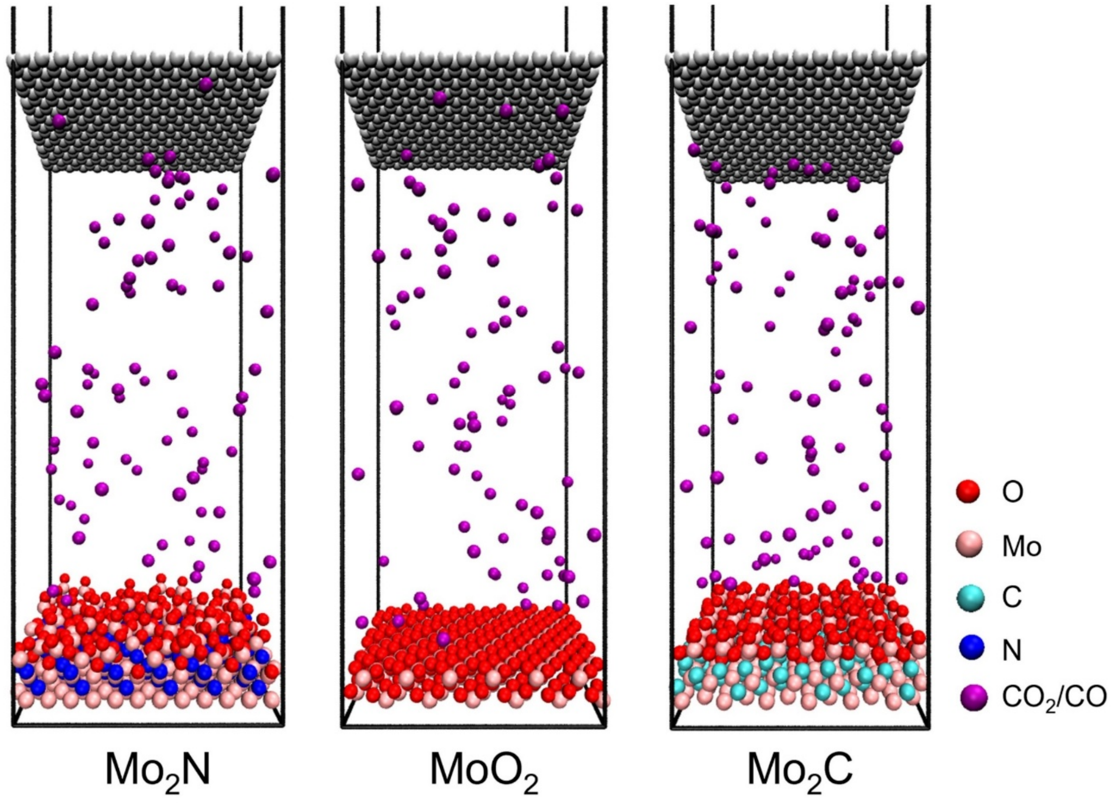

Supplementary Figure 12. MD simulation model of MoO_3/Mo_2N-C, MoO_3/MoO_2-C, and MoO_3/Mo_2C-C catalysts.

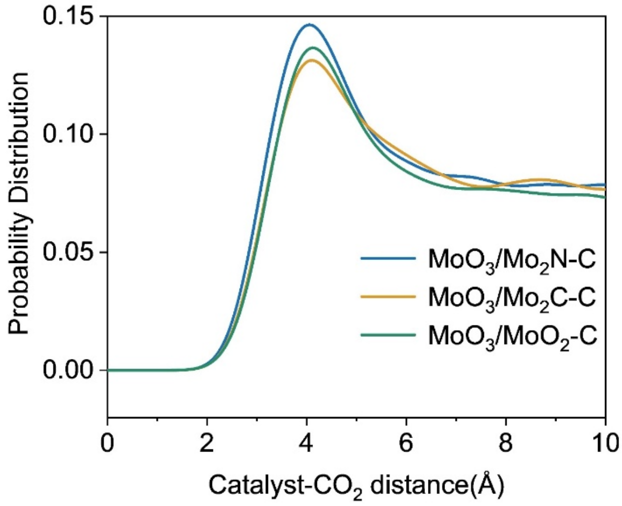

Supplementary Figure 13. Catalyst-CO_2 distance distribution within 1.5 nm for MoO_3/Mo_2N-C, MoO_3/MoO_2-C, and MoO_3/Mo_2C-C catalysts. (→ on x-axis: "Catalyst"

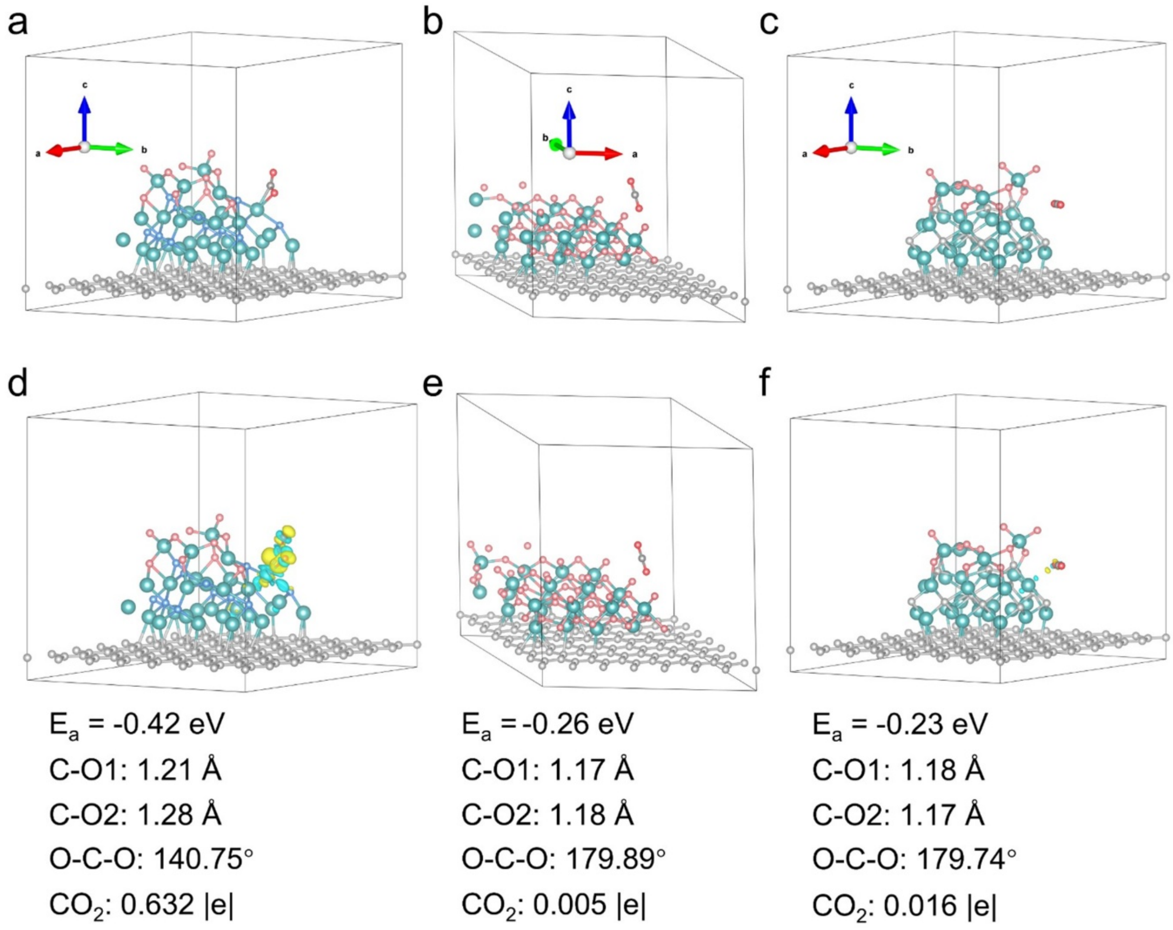

Supplementary Figure 14. a-c) Structure models and **d-f)** differential electron density plots of CO_2* on **a, d)** MoO_3/Mo_2N-C, **b, e)** MoO_3/MoO_2-C, and **c, f)** MoO_3/Mo_2C-C catalysts (cyan and yellow show charge consumption and accumulation, respectively, the cutoff of the density-difference isosurface is 0.004 e·Bohr^{-3}).

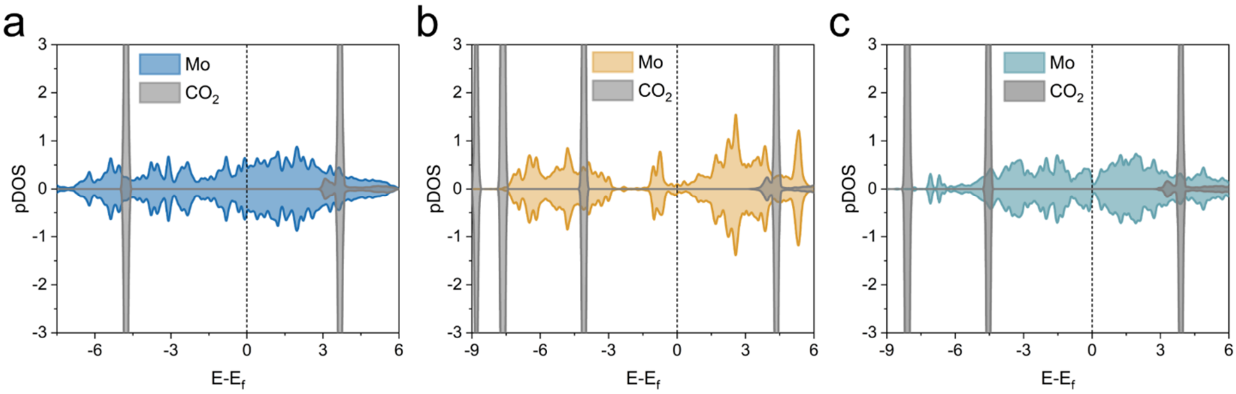

Supplementary Figure 15. PDOS analysis of Mo and CO_2 before adsorption for **a)** MoO_3/Mo_2N-C, **b)** MoO_3/MoO_2-C, **c)** MoO_3/Mo_2C-C catalysts.

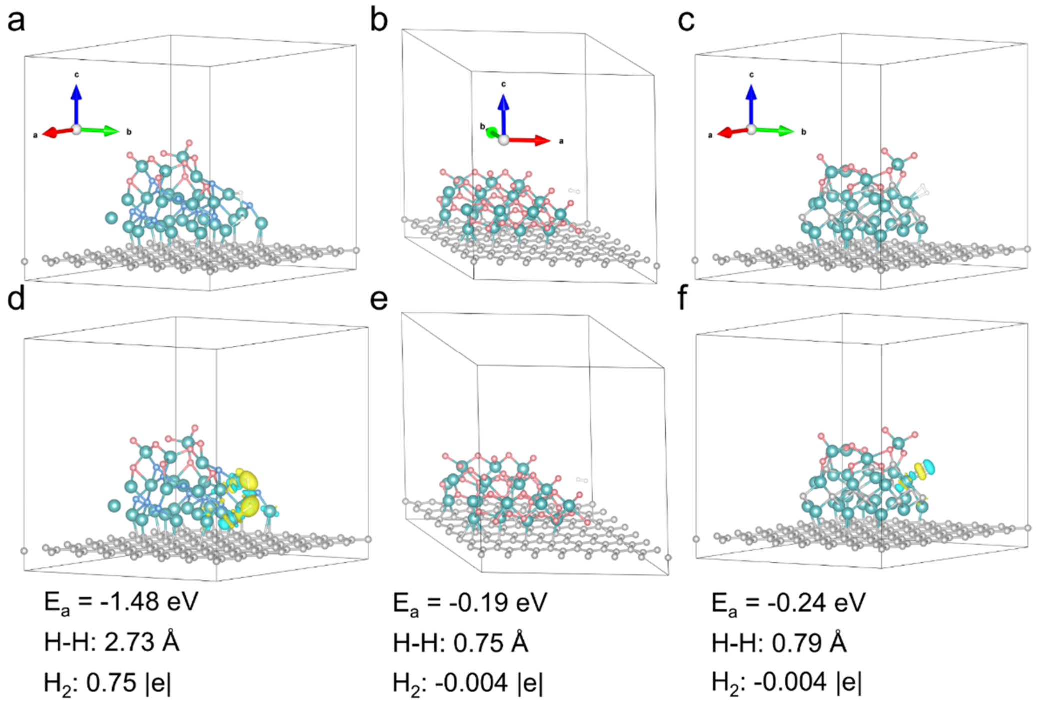

Supplementary Figure 16. a-c) Structure models and **d-f)** differential electron density plots of H_2* on **a, d)** MoO_3/Mo_2N-C, **b, e)** MoO_3/MoO_2-C, and **c, f)** MoO_3/Mo_2C-C catalysts (cyan and yellow show charge consumption and accumulation, respectively, the cutoff of the density-difference isosurface is 0.004 e·Bohr^{-3}).

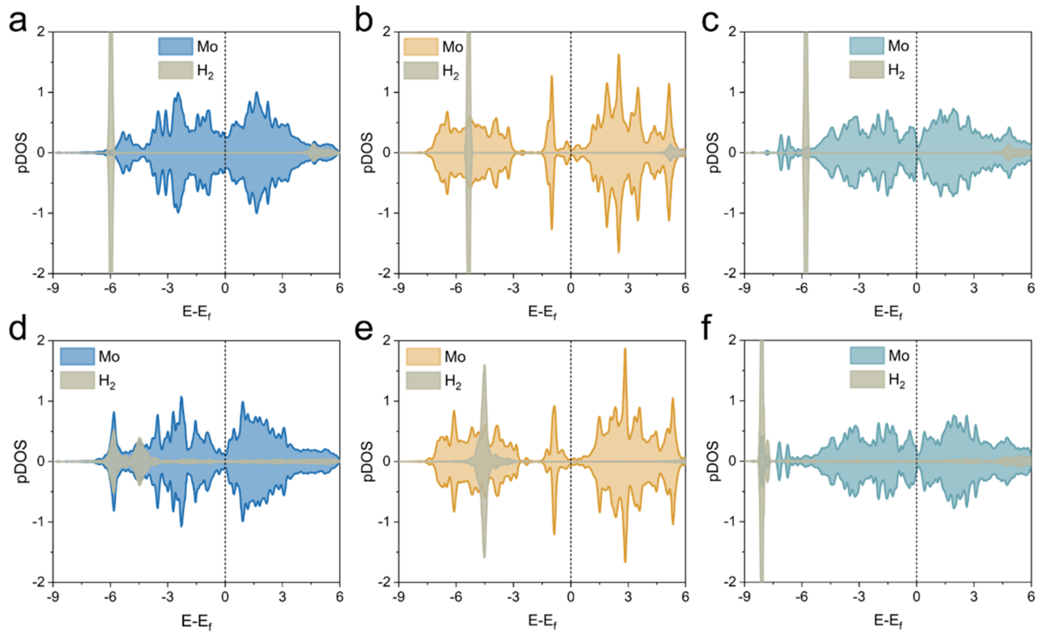

Supplementary Figure 17. PDOS analysis of Mo and H_2 **a-c)** before and **d-f)** after adsorption for **a, d)** MoO_3/Mo_2N-C, **b, e)** MoO_3/MoO_2-C, and **c, f)** MoO_3/Mo_2C-C catalysts.

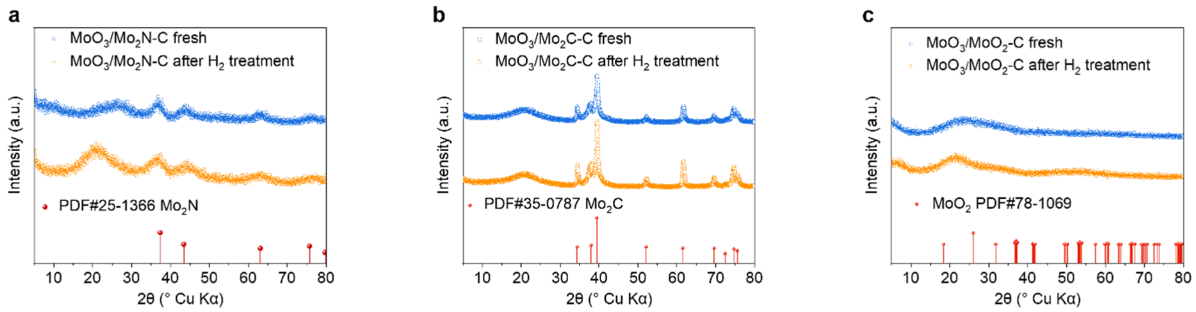

Supplementary Figure 18 a, b, c) XRD patterns of MoO3/Mo2N-C, MoO3/MoO2-C, and MoO3/Mo2C-C before and after H_2 treatment.

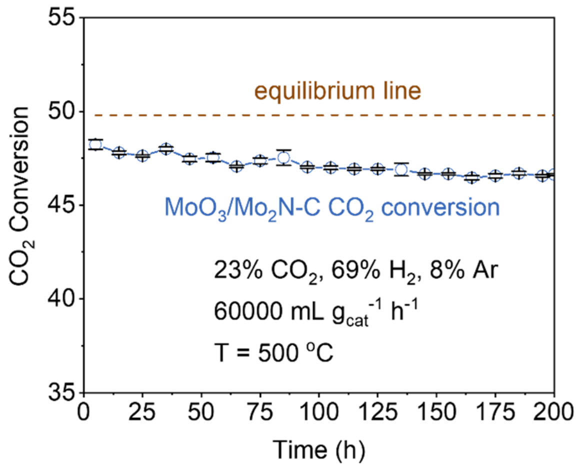

Supplementary Figure 19. Long-term stability test of MoO3/Mo2N-C under 60,000 mL $g_{cat}^{-1}h^{-1}$. (n = 3 independent experiments, data are presented as mean values ± SD)

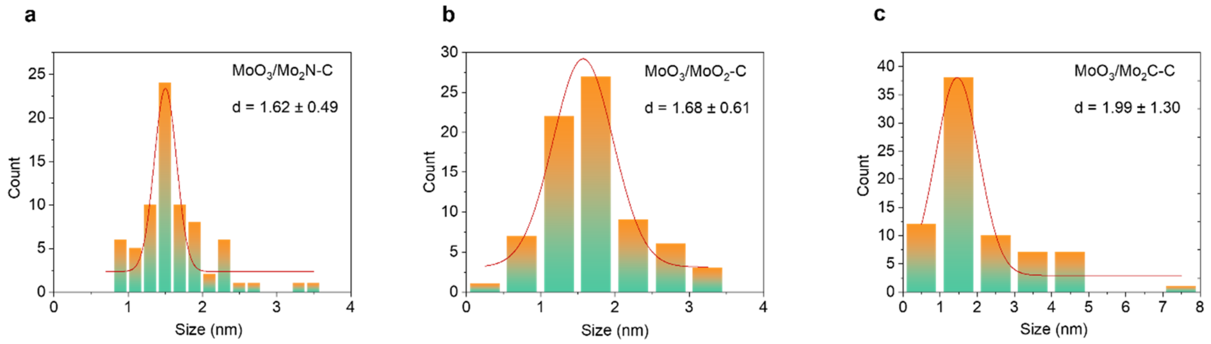

Supplementary Figure 20 STEM images-derived statistical analysis of the crystal sizes of **a)** MoO3/Mo2N-C, **b)** MoO3/MoO2-C, and **c)** MoO3/Mo2C-C.

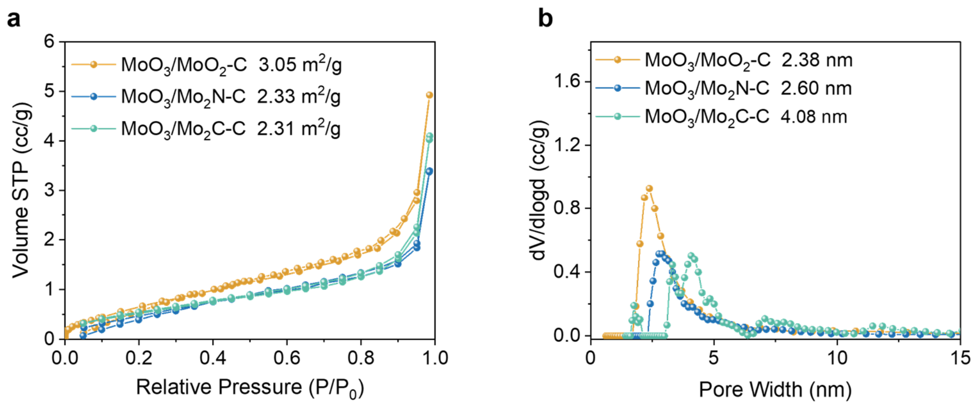

Supplementary Figure 21 a) N_2 adsorption/desorption isotherms of MoO_3/Mo_2N-C, MoO_3/MoO_2-C, and MoO_3/Mo_2C-C, **b)** Pore size distribution of MoO_3/Mo_2N-C, MoO_3/MoO_2-C, and MoO_3/Mo_2C-C.

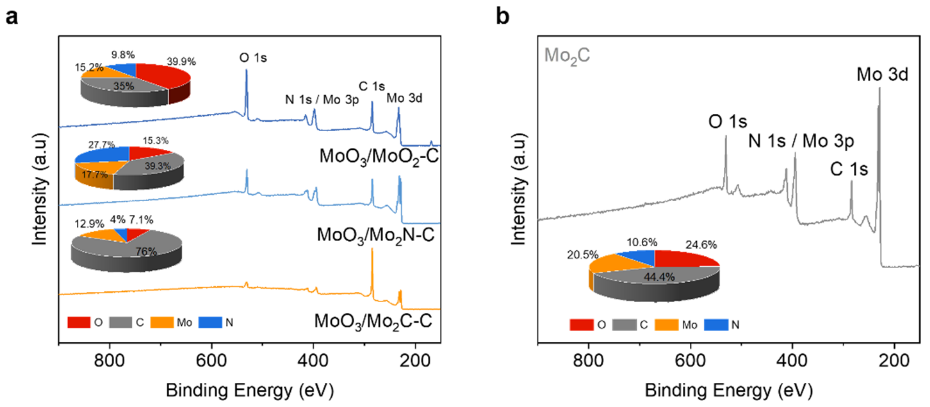

Supplementary Figure 22. a) XPS survey spectra and elemental content for MoO_3/Mo_2N-C, MoO_3/MoO_2-C, and MoO_3/Mo_2C-C, **b)** XPS survey spectra and elemental content for Mo_2C-C.

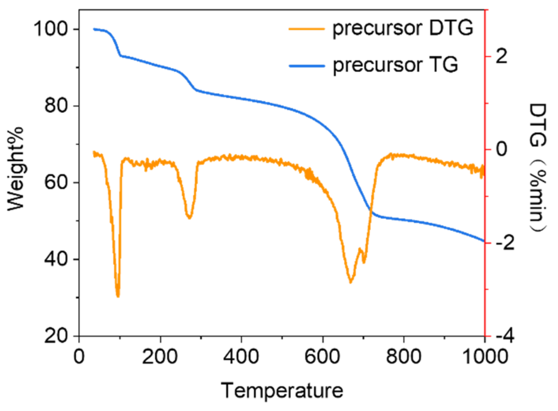

Supplementary Figure 23. Thermogravimetric analysis for the organic-polyoxometalate cMoO3/crystals.

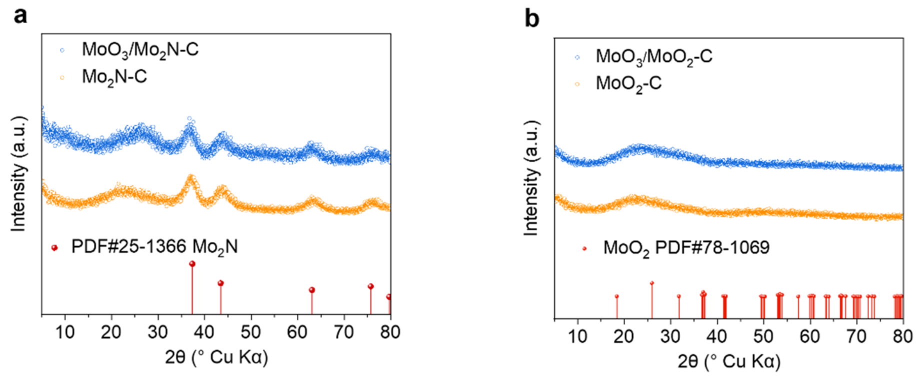

Supplementary Figure 24 a) XRD patterns of MoO3/Mo2N-C and Mo2N-C, **b)** XRD patterns of MoO3/MoO2-C and MoO2-C.

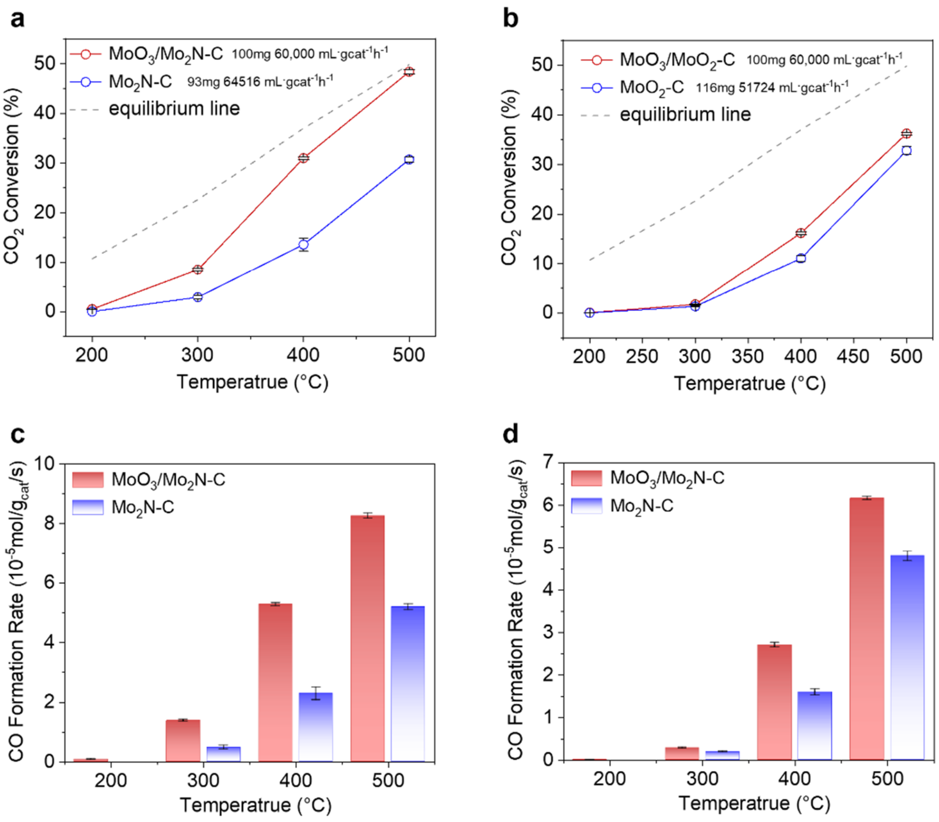

Supplementary Figure 25 a, b) CO_2 conversion at various temperatures, **c, d)** Catalytic reaction rates at various temperatures. (In a-d, n = 3 independent experiments, data are presented as mean values ± SD)

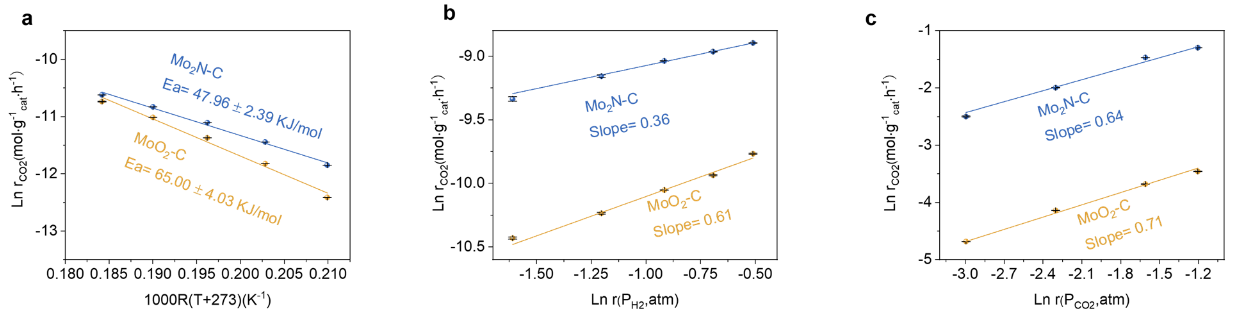

Supplementary Figure 26 a) Apparent activation energy (E_a), **b, c)** Kinetic orders of reactants (H_2 and CO_2) for Mo_2N-C and MoO_2-C catalysts. (In a-c, n = 3 independent experiments, data are presented as mean values ± SD)

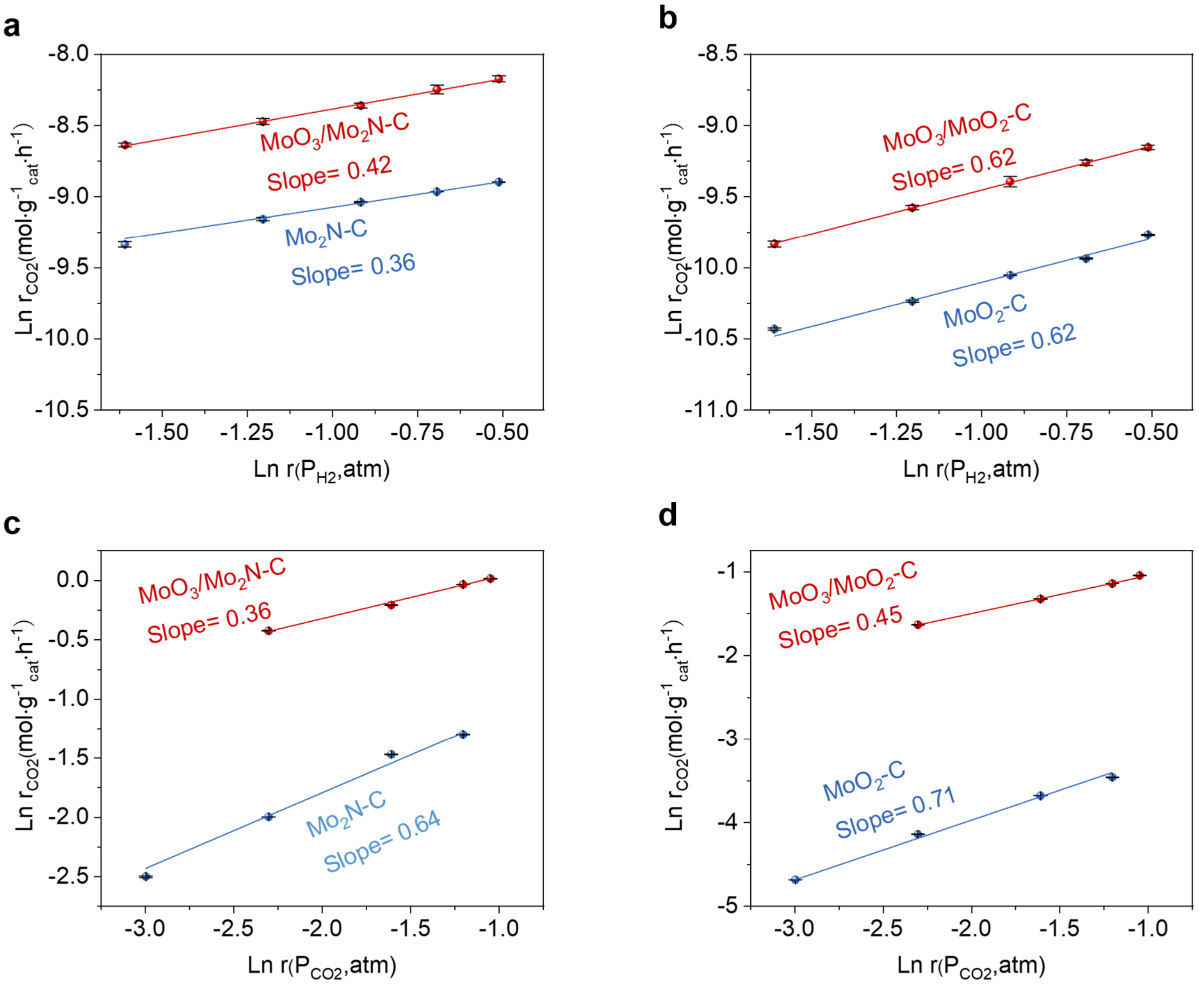

Supplementary Figure 27 a) Kinetic orders of H_2 for MoO_3/Mo_2N-C and Mo_2N-C catalysts, **b)** Kinetic orders of H_2) for MoO_3/MoO_2-C and MoO_2-C, **c)** Kinetic orders of CO_2 for MoO_3/Mo_2N-C and Mo_2N-C catalysts, **d)** Kinetic orders of CO_2 for MoO_3/MoO_2-C and MoO_2-C. (In a-d, n = 3 independent experiments, data are presented as mean values ± SD)

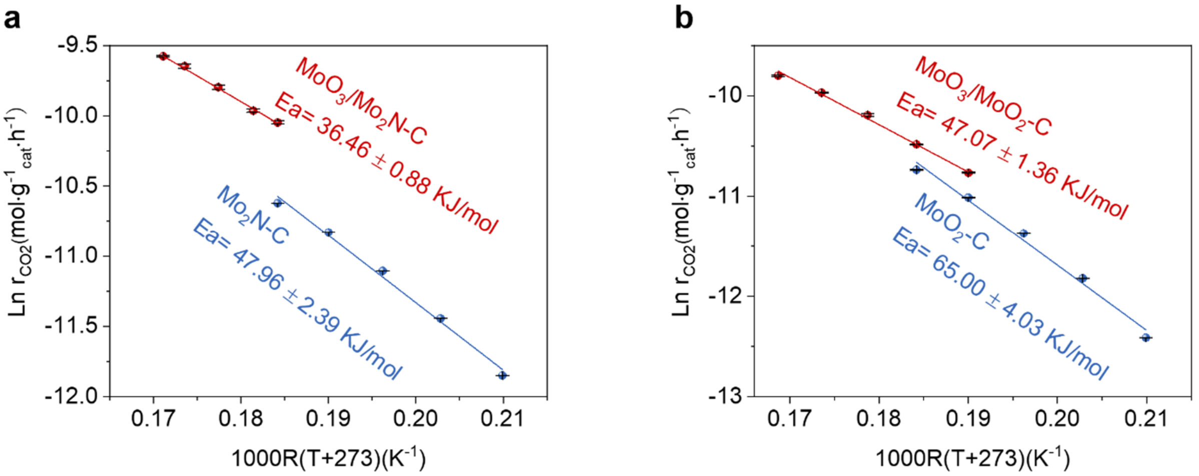

Supplementary Figure 28 a) E_a for MoO_3/Mo_2N-C and Mo_2N-C catalysts. **b)** E_a for MoO_3/MoO_2-C and MoO_2-C catalysts. (In a-b, n = 3 independent experiments, data are presented as mean values ± SD)

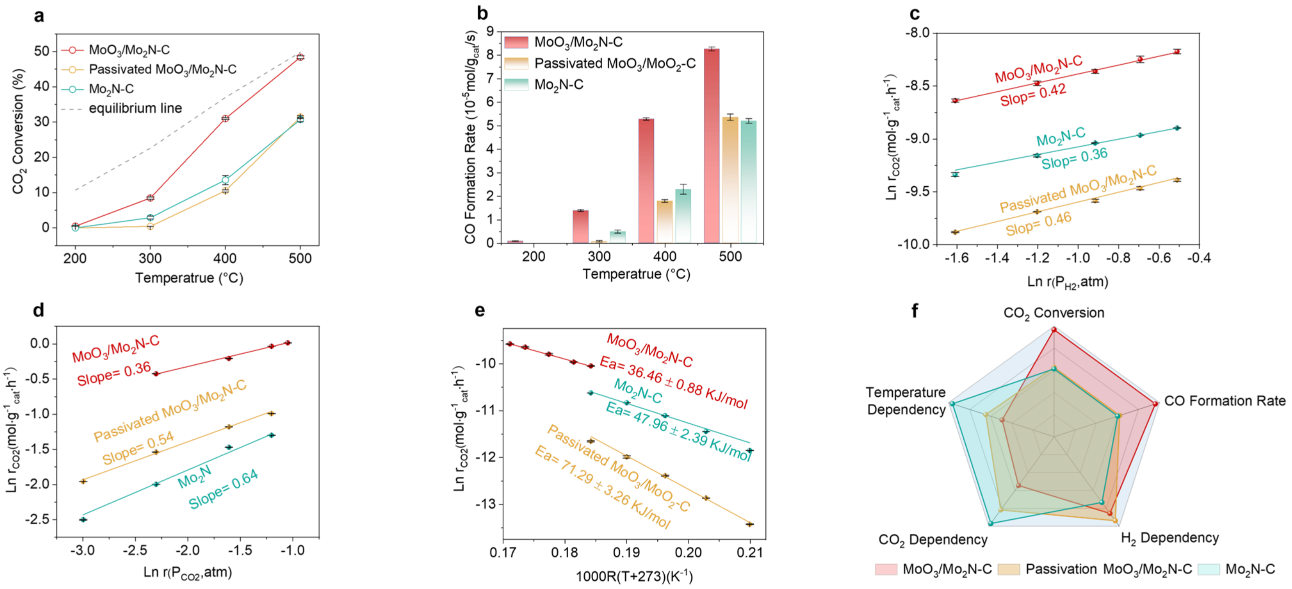

Supplementary Figure 29 a) CO_2 conversion at various temperatures, **b)** Catalytic reaction rates at various temperatures. **c)** Kinetic orders of H_2 for MoO_3/Mo_2N-C, passivated MoO_3/Mo_2N-C and Mo_2N-C catalysts, **d)** Kinetic orders of CO_2 for MoO_3/Mo_2N-C, passivated MoO_3/Mo_2N-C and Mo_2N-C catalysts. **e)** E_a for MoO_3/Mo_2N-C, passivated MoO_3/Mo_2N-C and Mo_2N-C catalysts. **b)** E_a for MoO_3/MoO_2-C and MoO_2-C catalysts. Performance comparison on the CO_2 conversion, reaction rates, Kinetic orders of H_2 and CO_2, and E_a of MoO_3/Mo_2N-C, passivated MoO_3/Mo_2N-C and Mo_2N-C catalysts. (In a-b, n = 3 independent experiments, data are presented as mean values ± SD)

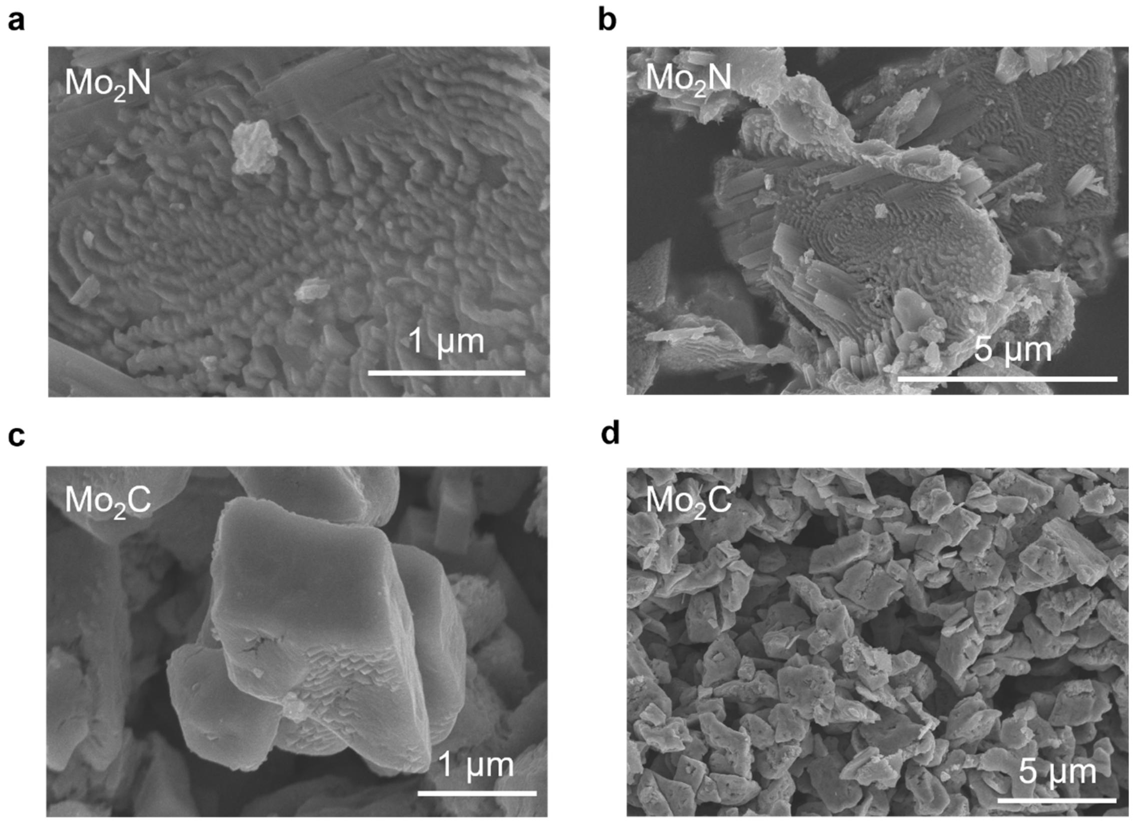

Supplementary Figure 30. SEM images of **a, b)** Mo_2N, **c, d)** Mo_2C prepared via ammonia and methane reduction.

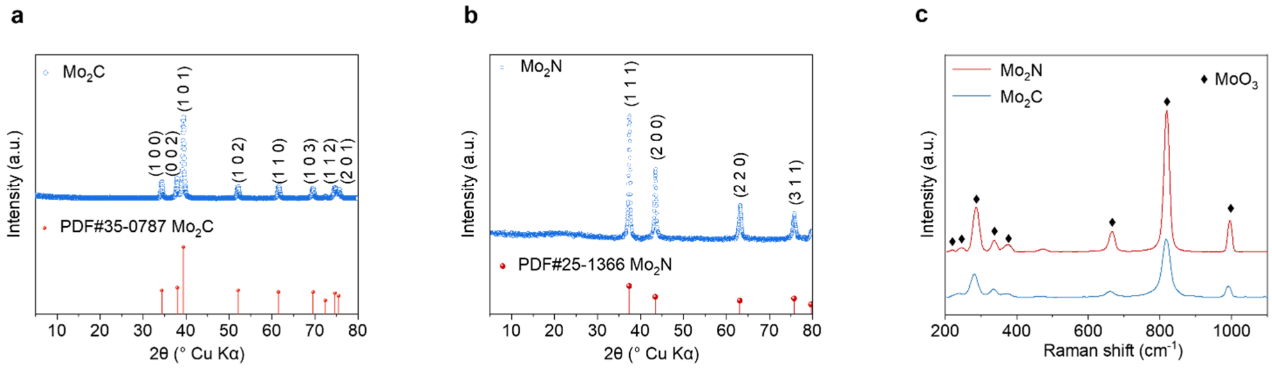

Supplementary Figure 31. XRD patterns of a) Mo_2C and b) Mo_2N, **c)** Raman spectra of Mo_2N and Mo_2C prepared via ammonia and methane reduction.

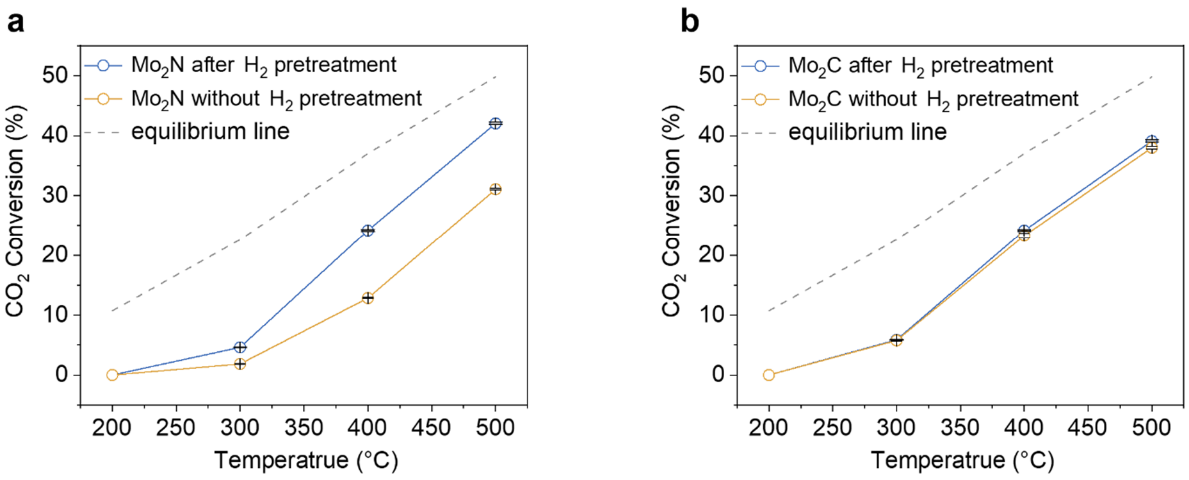

Supplementary Figure 32. a, b) Catalytic results for CO yield under different temperatures under 60,000 mL·$g_{cat}^{-1}h^{-1}$. (In a-b, n = 3 independent experiments, data are presented as mean values ± SD).

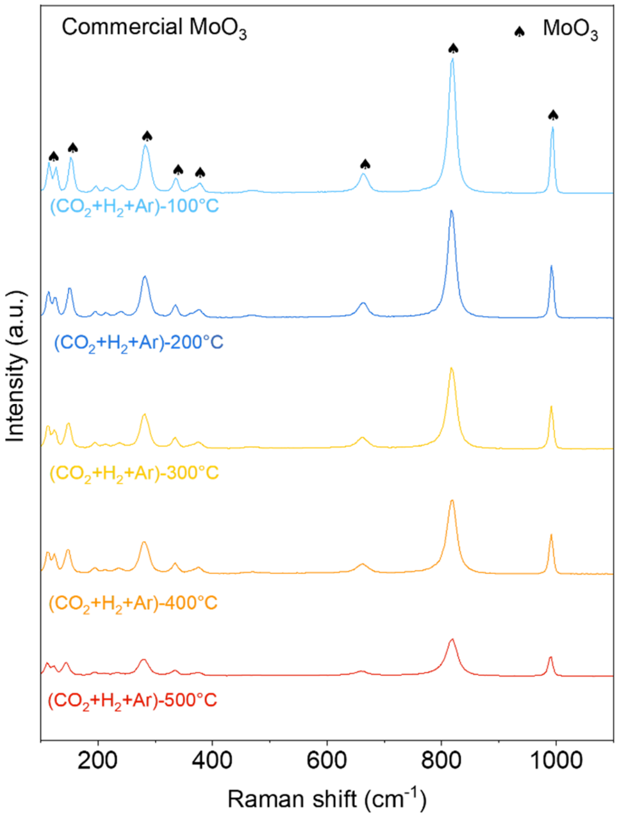

Supplementary Figure 33. In-situ Raman results of commercial-MoO_3 under the RWGS reaction.

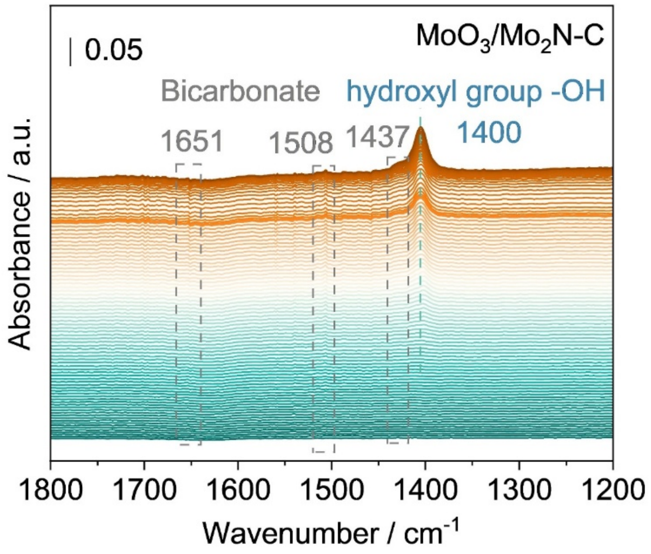

Supplementary Figure 34. In-situ DRIFT spectra of the RWGS reaction over MoO_3/Mo_2N-C at 25-500 °C (Pretreatment condition: 500 °C in H_2 diluted in Ar stream (1 mL min^{-1} for H_2 and 9 mL min^{-1} for Ar) for 1 h. Reaction conditions: 12 % H_2 and 4 % CO_2 in Ar (H_2/CO_2 molar ratio of 3) at 10 mL min^{-1}.

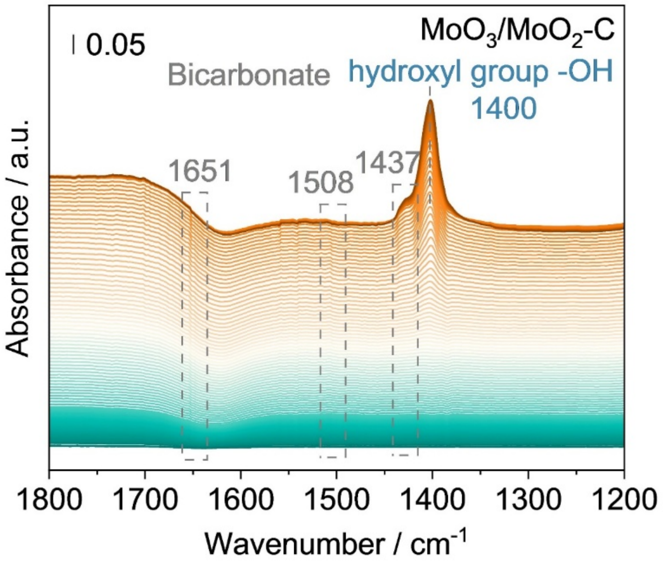

Supplementary Figure 35. In-situ DRIFT spectra of the RWGS reaction over MoO_3/Mo_2N-C, MoO_3/MoO_2-C, and MoO_3/Mo_2C-C at 25-500 °C (Pretreatment condition: 500 °C in H_2 diluted in Ar stream (1 mL min^{-1} for H_2 and 9 mL min^{-1} for Ar) for 1 h. Reaction conditions: 12 % H_2 and 4 % CO_2 in Ar (H_2/CO_2 molar ratio of 3) at 10 mL min^{-1}.

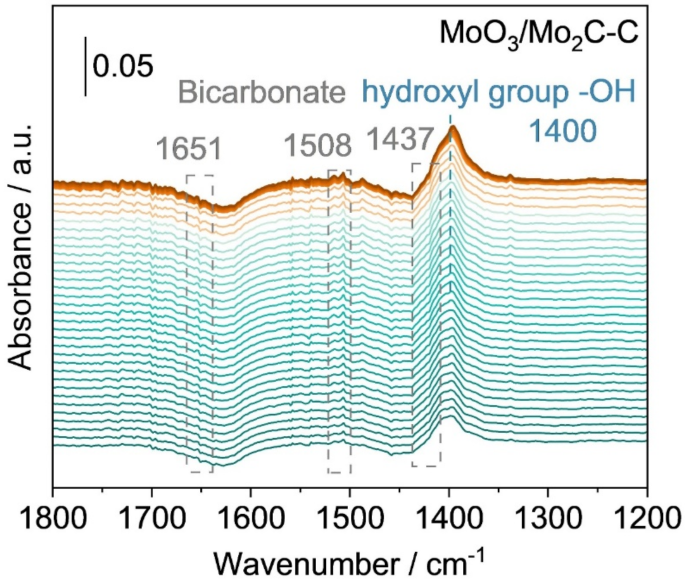

Supplementary Figure 36. In-situ DRIFT spectra of the RWGS reaction over MoO_3/Mo_2C-C at 25-500 °C (Pretreatment condition: 500 °C in H_2 diluted in Ar stream (1 mL min^{-1} for H_2 and 9 mL min^{-1} for Ar) for 1 h. Reaction conditions: 12 % H_2 and 4 % CO_2 in Ar (H_2/CO_2 molar ratio of 3) at 10 mL min^{-1}.

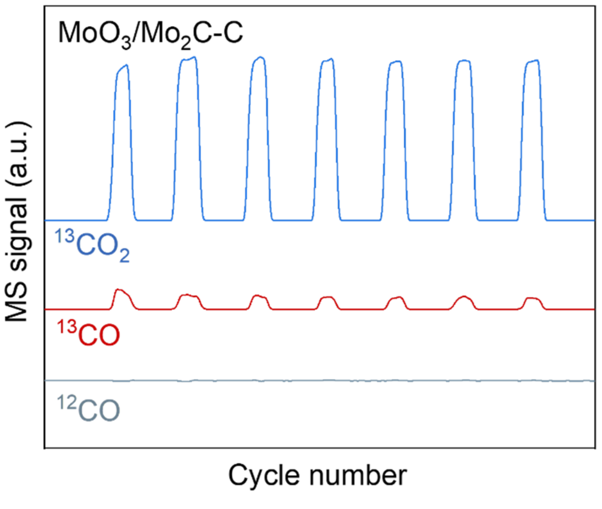

Supplementary Figure 37 Pulse experiments using isotope labelling in situ mass spectrometry with $^{13}CO_2$ for the MoO_3/Mo_2C-C catalysts at 500 °C.

Additionally, minor peaks are observed at 1437, 1508, and 1637 cm^{-1}, indicating a negligible presence of surface bicarbonate species across the three catalysts [1].

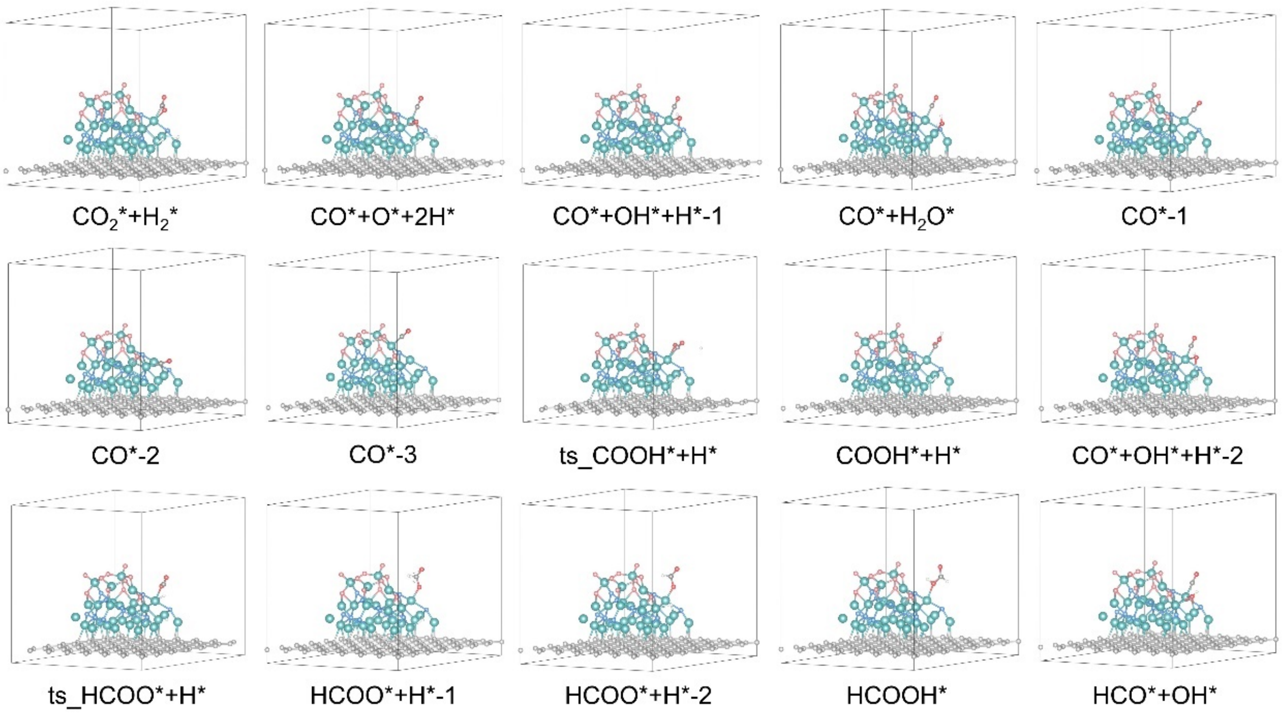

Supplementary Figure 38. Structure models of the three reaction routes (redox, carboxyl, and formate) on MoO_3/Mo_2N-C catalyst.

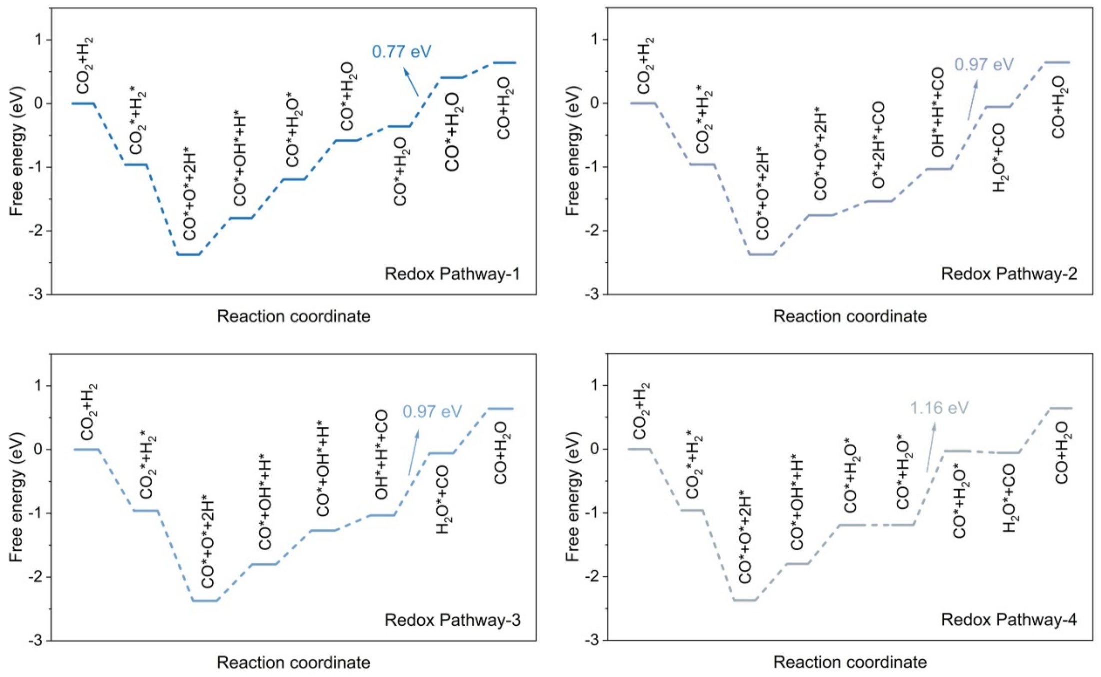

Supplementary Figure 39. Free energy profiles of the redox reaction pathways on MoO_3/Mo_2N-C.

Methods

Characterizations

Figures and artwork. Graphic elements in Figures 1a, 2 (c-e, h), 3g, 5g, Supplementary Figure 12, 38 were created using the opensource software Blender 3.6, VMD 1.9 and Inkscape 1.4.2, all distributed under the GNU General Public License (GPL).

Scanning electron microscope (SEM): The morphology of the as-prepared catalysts was observed by scanning electron microscopy (SEM, Hitachi Regulus 8220, Japan). All the carbonized materials were observed directly without gold coating. For the nonconductive precursors, the gold coating was deposited with a layer of about 1 nm.

Transmission electron microscopy (TEM): High-angle annular dark-field scanning TEM (HAADF-STEM) was performed on a probe-corrected JEM ARM 200F S/TEM (JEOL Japan).

X-ray photoelectron spectra (XPS): (XPS, ESCAL 250) are measured on a K-Alpha X-ray photoelectron spectrometer system (Thermo Scientific) with a Hemispheric 1800 dual-focus analyzer with a 128-channel detector.

Thermal gravimetric analysis (TGA): TGA was conducted in Ar conditions from room temperature to 1000°C with a ramp of 10°C /min.

X-ray diffraction (XRD): X-ray diffraction (XRD) pattern presents the crystal phase state via a DX-2700BH multipurpose X-ray diffractometer (Haoyuan Instrument) with Cu radiation at a voltage of 40 kV.

H_2 temperature-programmed reduction (H_2-TPR): Hydrogen temperature-programmed reduction (H_2-TPR) was carried out using a Builder PCSA-1000 instrument equipped with a thermal conductivity detector (TCD). The fresh catalysts (50 mg) were activated in Ar at 200 °C for 60 min, then cooled to room temperature, followed by heating the samples from room temperature to 500 °C with the ramping rate of 10 °C min^{-1} in the presence of 5% H_2/Ar flowing at the rate of 30 mL min^{-1}.

In-situ diffuse reflectance infrared Fourier transform spectroscopy (DRIFTS): All of the in-situ

DRIFTS spectra were collected by using a PerkinElmer Spectrum 3 FTIR spectrometer with a mercury cadmium telluride (MCT) detector cooled with liquid nitrogen. The treatment process of CO_2 on the fresh catalysts was investigated by in situ DRIFTS measurement at 500 °C. Before the in-situ DRIFTS measurement, ~20 mg of fresh catalysts were pretreated at 500 °C for 60 min under a 10% H_2/Ar mixed gas. The background spectra were collected under an N_2 atmosphere at 4 cm^{-1} resolution at 25-500 °C. After collecting the background spectrum, the mixed gas, which consisted of 2% CO_2/Ar and was introduced into the chamber. Continuous recording of the IR profiles was maintained for 5 min. As for the test under RWGS reaction conditions, after background acquisition, the reaction gas with 4% CO_2 /12% H_2 /84% N_2 is introduced into the in-situ chamber. All DRIFTS results were analyzed by using OPUS software.

XAFS (X-ray absorption fine spectroscopy) experiments: The extended X-ray absorption fine structure (EXAFS) measurements were carried out on the sample at the 5S1 X-ray absorption beamline of Aichi Synchrotron Radiation Center. This beamline adopted a double-bounce channel-cut Si (111) monochromator for mono-beam X-ray absorption spectroscopy. The end station is equipped with three ionization chambers and a seven-element SDD detector after the sample position for transmission and fluorescence mode X-ray absorption spectroscopy. The photon flux on the sample ranges from 3x1010 ~ 4x1010 photons/sec for X-ray energy from 5keV to ~ 9keV in low energy mode. The photon flux on the sample ranges from 1x1011 ~ 2.2x1010 photons/sec for X-ray energy from 7keV ~ 18keV in normal energy mode. The photon flux on the sample ranges from 2.3x1010 ~ 5x109 photons/sec for X-ray energy from 17keV ~ 22keV in high energy mode.

Raman spectroscopy: Ex situ and in-situ Raman spectra were acquired on a Raman micro-scope system (Xplora Plus, HORIBA FRANCE SAS) with laser excitation at 473 nm. The integration times of ex-situ and in-situ Raman spectra were 5 s and 90 s, respectively. For the in-situ Raman, the micro-Raman reaction cell (Beijing Scistar Technology Co. Ltd) equipped with a quartz window has a heating module that controls the test temperature. Two kinds of programs were conducted as follows.

(i) Room-temperature treatments. MoO_3/Mo_2N-C, MoO_3/MoO_2-C, and MoO_3/Mo_2C-C catalysts were purged by 5% H_2/Ar subsequently at RT. The Raman spectra were collected at RT.

(ii) The MoO_3/Mo_2N-C, MoO_3/MoO_2-C, and MoO_3/Mo_2C-C catalysts and commercial MoO_3 were pretreated by 5% H_2/Ar at 500 °C for 60 min. After cooling to room temperature, the gas

flow was switched to RWGS reaction gas (23% CO_2, 69% H_2, 8% Ar). The Raman spectra were collected at 100, 200, 300, 400, and 500 °C, respectively.

Computational Methods. All theoretical calculations were performed using the DFT method, as implemented in the Vienna ab initio simulation package (VASP)[2, 3, 4]. The core electrons were described using the spin-polarized projector augmented wave (PAW) method [5], and the electron exchange and correlation energy were treated within the generalized gradient approximation in the Perdew-Burke-Ernzerhof functional (GGA-PBE)[6]. The valence states of all atoms were expanded in a plane-wave basis set with a cutoff energy of 500 eV. The convergence criteria for the electronic self-consistent iteration and force were set to 10^{-5} eV and 0.02 eV/Å with a Gamma-centered 2 × 2 × 1 K-points. Denser 3 × 3 × 1 K-points were used for the density of states (DOS) computations. Van der Waals (vdW) corrections with zero damping DFT-D3 method of Grimme were used in all structures [7, 8]. The Mo_2N (111), MoO_2 (010), and Mo_2C (001) slabs were modeled. During the simulation, the bottom layers were fixed while the top layers were kept fully relaxed. The slab model was constructed with a vacuum layer of 15 Å in the z direction to avoid the interaction between neighboring images. The charge density differences were evaluated using the formula $\Delta\rho = \rho_{A+B} - \rho_A - \rho_B$, where ρ_X is the electron density of X. Atomic charges were computed using the atom-in-molecule (AIM) scheme proposed by Bader[9, 10].

Crystal orbital Hamilton population (COHP) analysis was performed with the LOBSTER4.1.0 package, which reconstructs the orbital-resolved wave functions via the projection of the delocalized PAW to localized atomic-like basis sets[11, 12, 13]. Basis sets given by pbeVaspFit2015 with additional functions fitted to atomic VASP GGA-PBE wavefunctions were used[14].

The climbing-image nudged elastic band (CI-NEB) and Dimer algorithms were utilized for transition state searches[15, 16], with the atomic force criterion relaxed to 0.05 eV/Å. It was confirmed that transition-state structures possessed only one imaginary frequency in the direction of the reaction. The activation energies were calculated by the following equation:

$$\Delta E_{TS} = E_{TS} - E_{IS} \quad (4)$$

Where E_{TS} is the transition state energy, and E_{IS} is the energy of the initial state.

The adsorption energy was defined by using the following expression:

$$E_{ads} = E_{(surf+mol)} - E_{(surf)} - E_{(mol)} \qquad (5)$$

Where $E_{(surf)}$ and $E_{(mol)}$ are the energies of substrates and isolated molecules, respectively, and $E_{(surf+mol)}$ represents the energy of the combined systems upon adsorption. This means that a negative E_{ads} value corresponds to exothermic adsorption. The models used in DFT calculations were constructed based on the comprehensive experimental characterization results. First, Mo_2N, MoO_2, and Mo_2C are identified from HAADF-STEM and XRD. Subsequently, Raman spectra further reveal that a few layers of MoO_3 are present on the three catalysts, which are thin enough that no MoO_3 diffraction peaks or lattice fringes are detected in XRD and HAADF-STEM characterization. Finally, different surface reconstructions during the RWGS process were observed by in-situ Raman spectroscopy, which guided the construction of DFT models under catalytic reaction conditions.

Molecular dynamics simulation. An all-atomic molecular model was constructed to explore the effect of different catalyst surfaces on adsorption. As shown in Supplementary Figs. 14, 15, the catalysts were positioned at the bottom of the simulation box, and 90 gas molecules (CO or CO_2) were randomly placed above the catalyst surface. A plate was placed at the top to prevent the gas molecules from escaping.

The whole simulation box is about 3.2×3.5×15 nm^3. Periodic boundary conditions were applied to all three directions. The catalysts were assumed to be rigid with nonbonding interactions. $\sum 4\varepsilon_{ij}\left[\left(\frac{\sigma_{ij}}{r_{ij}}\right)^{12} - \left(\frac{\sigma_{ij}}{r_{ij}}\right)^{6}\right] + \sum \frac{q_i q_j}{4\pi\varepsilon_0 r_{ij}}$, where ε_{ij} and σ_{ij} are the well and collision diameter of the Lennard-Jones (LJ) potential, r_{ij} is the distance between atoms i and j, q_i is the atomic charge of atom i, and ε_0 is the permittivity of a vacuum. The LJ potential parameters were adopted from the universal force field (UFF)[17]. The atomic charges of the electrocatalysts were generated using DDEC atomic charge [18]. CO_2 and CO models were adopted from the literature[19, 20].

The electrostatic interactions were calculated using the Particle-Mesh Ewald method, while the LJ interactions were calculated using a cutoff of 1.2 nm. The temperature was maintained at 773 K using a v-rescale scheme, and the time step was 1 fs. After equilibration, a constant number of particles, volume, and temperature (NVT) ensemble was used for output simulation. The NVT simulation duration was 10 ns. All the MD simulations were

performed using GROMACS 2021 [21] and the model was visualized by VMD 1.9 [22].

Supplementary References:

1. Szanyi J, Kwak JH. Dissecting the steps of CO2 reduction: 1. The interaction of CO and CO_2 with γ-Al2O3: an in situ FTIR study. *Phys. Chem. Chem. Phys.* **16**, 15117-15125 (2014).

2. Kresse G, Furthmiiller J. Efficiency of ab-initio total energy calculations for metals and semiconductors using a plane-wave basis set. *Comput Mater Sci* **6**, 15-50 (1996).

3. Kresse G, Furthmuller J. Efficient iterative schemes for ab initio total-energy calculations using a plane-wave basis set. *Phys Rev B* **54**, 11169-11186 (1996).

4. Kresse G, Hafner J. Ab initiomolecular-dynamics simulation of the liquid-metal–amorphous-semiconductor transition in germanium. *Phys Rev B* **49**, 14251-14269 (1994).

5. Blochl PE. Projector augmented-wave method. *Phys Rev B* **50**, 17953-17979 (1994).

6. Perdew JP*, et al.* Atoms, molecules, solids, and surfaces: Applications of the generalized gradient approximation for exchange and correlation. *Phys Rev B* **46**, 6671-6687 (1992).

7. Klimeš J, Bowler DR, Michaelides A. Chemical accuracy for the van der Waals density functional. *J Phys: Condens Matter* **22**, 022201 (2010).

8. Klimeš J, Bowler DR, Michaelides A. Van der Waals density functionals applied to solids. *Phys Rev B* **83**, 195131 (2011).

9. Bader RFW. A quantum theory of molecular structure and its applications. *Chem Rev* **91**, 893-928 (1991).

10. Tang W, Sanville E, Henkelman G. A grid-based Bader analysis algorithm without lattice bias. *J Phys: Condens Matter* **21**, 084204 (2009).

11. Deringer VL, Tchougréeff AL, Dronskowski R. Crystal Orbital Hamilton Population (COHP) Analysis As Projected from Plane-Wave Basis Sets. *J Phys Chem A* **115**, 5461-5466 (2011).

12. Dronskowski R, Blochl PE. Crystal Orbital Hamilton Populations (COHP). Energy-Resolved Visualization of Chemical Bonding in Solids Based on Density-Functional Calculations. *J Phys Chem* **97**, 8617-8624 (1993).

13. Maintz S, Deringer VL, Tchougréeff AL, Dronskowski R. Analytic projection from plane-wave

and PAW wavefunctions and application to chemical-bonding analysis in solids. *J Comput Chem* **34**, 2557-2567 (2013).

14. Maintz S, Deringer VL, Tchougréeff AL, Dronskowski R. LOBSTER: A tool to extract chemical bonding from plane-wave based DFT. *J Comput Chem* **37**, 1030-1035 (2016).

15. Henkelman G, Uberuaga BP, Jónsson H. A climbing image nudged elastic band method for finding saddle points and minimum energy paths. *The Journal of Chemical Physics* **113**, 9901-9904 (2000).

16. Henkelman G, Jónsson H. A dimer method for finding saddle points on high dimensional potential surfaces using only first derivatives. *The Journal of Chemical Physics* **111**, 7010-7022 (1999).

17. Rappé AK, Casewit CJ, Colwell K, Goddard III WA, Skiff WM. UFF, a full periodic table force field for molecular mechanics and molecular dynamics simulations. *Journal of the American chemical society* **114**, 10024-10035 (1992).

18. Limas NG, Manz TA. Introducing DDEC6 atomic population analysis: part 4. Efficient parallel computation of net atomic charges, atomic spin moments, bond orders, and more. *RSC advances* **8**, 2678-2707 (2018).

19. Harris JG, Yung KH. Carbon dioxide's liquid-vapor coexistence curve and critical properties as predicted by a simple molecular model. *The Journal of Physical Chemistry* **99**, 12021-12024 (1995).

20. Sirjoosingh A, Alavi S, Woo TK. Grand-canonical Monte Carlo and molecular-dynamics simulations of carbon-dioxide and carbon-monoxide adsorption in zeolitic imidazolate framework materials. *The Journal of Physical Chemistry C* **114**, 2171-2178 (2010).

21. Abraham MJ*, et al.* GROMACS: High performance molecular simulations through multi-level parallelism from laptops to supercomputers. *SoftwareX* **1**, 19-25 (2015).

22. Humphrey W, Dalke A, Schulten K. VMD: visual molecular dynamics. *Journal of molecular graphics* **14**, 33-38 (1996).
